# A modular and reusable model of epithelial transport in the proximal convoluted tubule

**DOI:** 10.1371/journal.pone.0275837

**Published:** 2022-11-10

**Authors:** Leyla Noroozbabaee, Pablo J. Blanco, Soroush Safaei, David P. Nickerson

**Affiliations:** 1 Auckland Bioengineering Institute, University of Auckland, Auckland, New Zealand; 2 National Laboratory for Scientific Computing, Petrópolis, Brazil; Emory University School of Medicine, UNITED STATES

## Abstract

We review a collection of published renal epithelial transport models, from which we build a consistent and reusable mathematical model able to reproduce many observations and predictions from the literature. The flexible modular model we present here can be adapted to specific configurations of epithelial transport, and in this work we focus on transport in the proximal convoluted tubule of the renal nephron. Our mathematical model of the epithelial proximal convoluted tubule describes the cellular and subcellular mechanisms of the transporters, intracellular buffering, solute fluxes, and other processes. We provide free and open access to the Python implementation to ensure our multiscale proximal tubule model is accessible; enabling the reader to explore the model through setting their own simulations, reproducibility tests, and sensitivity analyses.

## I. Introduction

Kidneys are vital organs and play an essential role in the overall homeostasis of the body in mammals. A human kidney is typically composed of one million nephrons, which are the primary functional unit of the kidney. The nephron consists of the renal corpuscle and the renal tubule. A renal tubule is a tubular structure composed of a single layer of epithelial cells divided into various functional segments. Each segment of the nephron has specific functions in the regulation of blood and urine composition. The proximal convoluted tubule (PCT) is considered one of the most significant functional segments in the nephron and a key contributor to pathologies such as hypertension and diabetes.

To gain a deeper understanding of the mechanisms and investigate any hypothesis regarding the underlying physiopathological conditions, such as hypertension, diabetes, or other kidney diseases, a virtual nephron model is an invaluable tool. A model like this can serve as a virtual laboratory, ideally be inexpensive to run, and should target the minimisation of animal experiments.

Many models of epithelial transport have been published that would be relevant and useful to integrate into a virtual nephron model, but often there is insufficient information in the literature to enable readers to reproduce the published observations and predictions, thus making their reuse in novel studies time consuming and resource intensive. Furthermore, as models evolve over time, any given version of a model is usually designed to investigate specific hypotheses. The various instances of a given model, or a family of models, are therefore inconsistent and require the reader to search the literature to discover or infer the modifications required to integrate the models and successfully reproduce published results. Such modifications, for example, may be as trivial as changes in parameter values or alterations of physical units, or as complex as specific assumptions made or mathematical formulations chosen. These problems hamper efforts to develop a virtual nephron model.

To help address this problem, we introduce here an integrative mathematical model of the PCT that reproduces the capabilities of existing renal models. We used a modular approach to build our model, which we believe will improve reusability while ensuring that the segment function predicted by the source models can be reproduced. At the same time, this approach is sufficiently flexible and configurable to be able to adapt to different segments or epithelia. This model has been implemented in Python and is freely available under an open-source and permissive license at https://github.com/iNephron/W-PCT-E. Our long-term goal is to make this resource available in a more reproducible and reusable form via the CellML standard [1, 2] and the bond graph approach [3–8] that ensures energy conservation as well as mass and charge conservation across different physical systems (biochemical, electrical, mechanical and metabolic). However, to bring this work into a consistent and unified mathematical framework and to understand the various parameter combinations and manipulations that have been used in a series of publications spanning 30+ years, we first implement in a Python scripting environment with clearly defined parameters and consistent physical units throughout.

This work would not have been possible without the comprehensive epithelial and tubular modelling published by Alan Weinstein, and colleagues, examining many aspects of renal function [9–16, for example]. Our implementation is derived from information available in the literature, supplemented with additional equations and parameter values discovered in Weinstein’s original implementation available at https://github.com/amweins/kidney-models-amw and also the work of other researchers who have adopted and confirmed the Weinstein model, such as [17–19] (with accompanying implementation available at https://github.com/Layton-Lab/nephron). As mentioned above, our goal here is to make this resource available to the renal modelling community in a manner that enhances reproducibility and reusability.

## II. Design and implementation

**Fig 1:**
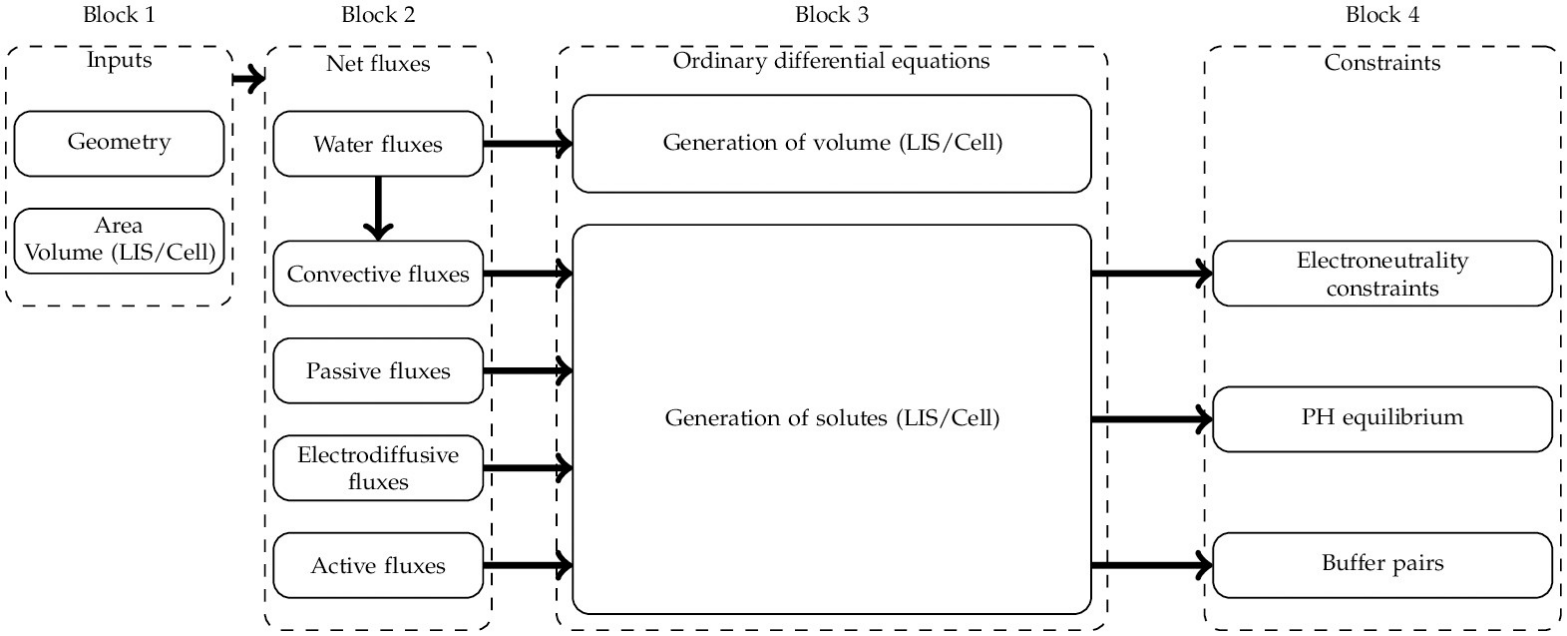
The logical blocks of the epithelial model implementation. The first block indicates the geometrical inputs (see Section vii). The second block shows the intra-epithelial fluxes; these fluxes will be involved in the mass conservation equations. The third block indicates the total mass conservation differential equations present in the epithelial model. The differential equations are updated by the system’s buffer pairs, pH equilibrium and electroneutrality to add more constraints to the system. The sequential placement of the blocks is not related to a procedural description of the model simulation but helps to understand the model construction.

In this work, we follow the comprehensive PCT epithelial model collectively presented in Weinstein et al. [9–13] which we refer to here as the W-PCT-E. The W-PCT-E consists of four logical blocks, see Fig 1. The first one provides the geometrical definitions of the system and the equations of the selected components, such as cell volume, lateral intercellular volume, basement membrane area, and epithelial thickness. These parameters are variable, whereas the rest of the geometrical components, such as membrane area for the other regions, are constant (see Section vii). The second block defines five different intra-epithelial fluxes: water fluxes, convective fluxes, passive fluxes, coupled solute fluxes, and active (metabolically dependent) fluxes. The output from the first block is coupled to the input of the second block, plus the specification of the membrane types and solutes that appear in the chemical reactions. The outcome of the second block are the total membrane solute fluxes and membrane water fluxes.

In the third block, the W-PCT-E model enforces the mass conservation of the system through differential equations within each compartment (or each membrane) with the total solute fluxes and water fluxes as input for each membrane.

In the final block, the W-PCT-E model applys more constraints by defining buffer pairs, pH equilibrium, and electroneutrality of the system.

## III. Model illustrations

**Fig 2:**
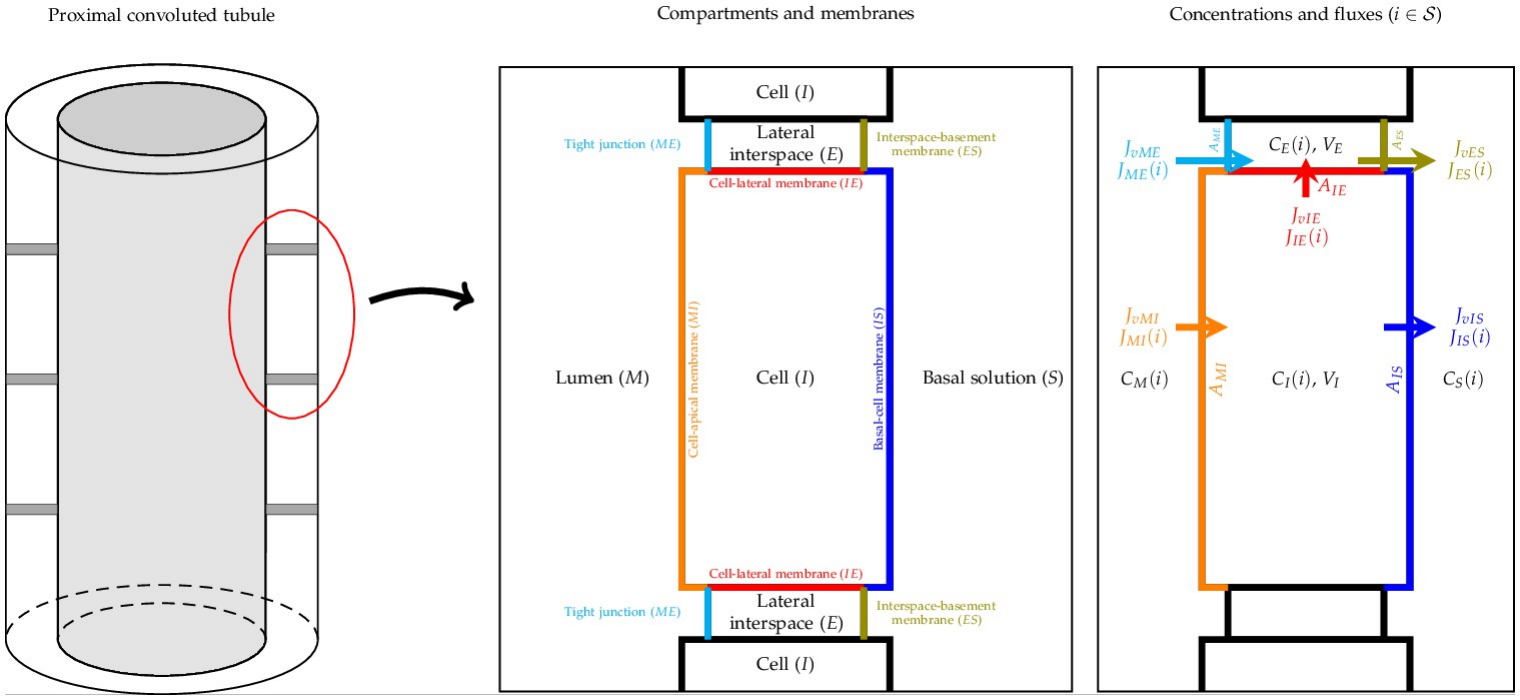
Schematic representation of the proximal convoluted tubule (PCT) epithelium, consisting of cell and lateral intercellular space, and a tubule model, in the way lumen is lined by epithelium. Intra-epithelial fluxes are designated *J*_*αβ*_(*i*), where the subscript *αβ* refers to different membranes and (*i*) refers to a specific solute.

Here, we define the various components in the modelling framework in the W-PCT-E system, including all equations, definitions, and assorted tables of constants and variables that appear in the epithelial model’s compartments. In addition, we introduce the ingredients present in our Python code and, whenever possible, the provenance of the parameter values. The comprehensive W-PCT-E model consists of cellular and lateral intercellular compartments between luminal and peritubular solutions. Fig 2 shows a schematic representation of PCT epithelium and features both configurations, in which cellular and lateral intercellular (LIS) compartments line the tubule lumen. Within each compartment, the concentration of species (i) is designated *C*_*α*_(*i*), where *α* is lumen (M), lateral interspace (E), cell (I) or basal solution (S). Separating membranes are combinations of letters such that luminal cell membrane (lumen-cell membrane, MI), tight junction (ME), cell-lateral membrane (IE), interspace basement membrane (ES), or cell-basal membrane (IS). The order of the two letters indicates the positive direction of the mass flow. *J*_*αβ*_ and *J*_*ναβ*_ represent the solute flux and water flux, respectively, through the corresponding membrane; A is the corresponding membrane surface area; V is the volume; E is the trans-epithelial potential difference. Symbols are defined in the following sections as introduced by Weinstein et al. [12] in the epithelial PCT model. Intra-epithelial fluxes are designated *J*_*αβ*_(*i*), where *αβ* refers to the different membranes. Models can include many different solutes in various compartments. In this paper, according to [12], the model consists of 15 solutes, namely, Na^+^, K^+^, Cl^−^, HCO_3_^−^, CO_2_, H_2_CO_3_, HPO_4_^2−^, H_2_PO_4_^−^, Urea, NH_3_, NH_4_^−^, H^+^, HCO_2_^−^, H_2_CO_2_, and glucose, as well as two impermeant species within the system; a nonreactive anion and a cytosolic buffer. The solutes considered in a specific simulation experiment can vary, leading to the dynamics of some of the solutes being ignored under certain conditions. There are 14 transporters (symporters, antiporters, complex transporters, and ATPases) that produce electrochemical fluxes in the W-PCT-E model.

Overall, there are six symporters on different membranes; two on the lumen-cell (MI): Sodium-Glucose (SGLT) and Sodium-Phosphate (NPT1), two on the cell-basal (IS): Potassium-Chloride (KCC4) and Sodium-Bicarbonate (NBC1), and two on the cell-lateral (IE): Potassium-Chloride (KCC4) and Sodium-Bicarbonate (NBC1). It is important to note that the cell-basal and cell-lateral membranes share a similar layout.

There are four antiporters on the lumen-cell membrane: Chloride/Bicarbonate (AE1), Chloride/Formate, Sodium/Hydrogen, and Sodium/Ammonium (NHE3).

There are two complex exchangers on the cell-lateral membrane: Sodium-Bicarbonate/Chloride (NCBE) and one on the cell-basal membrane: Sodium-Bicarbonate/Chloride (NCBE). The Na/K pumps (NaK-ATPase) are located in both cell-basal and cell-lateral membranes. H-pumps (H-ATPase) are located in the lumen-cell membrane, regulating the pH in the W-PCT-E model. We noticed two different approaches to translate the behaviour of sodium/hydrogen antiporter in Weinstein’s work. In the first approach, Weinstein et al. used the equivalent definition of two antiporters: Sodium/Hydrogen and Sodium/Ammonium [11]. In the second approach, reported in [12], the authors used a detailed model of the Na^+^/H^+^ antiporter from [20].

### i. State equations

First, we introduce the sets of compartments (C) and membranes (M) indexes

$$\begin{array}{c} C=\{I,E,S,M\}, \end{array}$$ (1)

$$\begin{array}{c} M=\{IE,IS,MI,ES,ME\}. \end{array}$$ (2)

Indexes in M might appear interchanged in some flux definitions, which means that the flux sign must be inverted. Now, we introduce the following set of solutes

$$\begin{array}{c} S=\{{\text{Na}}^{+},{\text{K}}^{+},{\text{Cl}}^{-},{{\text{HCO}}_{3}}^{-},{\text{CO}}_{2},{\text{H}}_{2}{\text{Co}}_{3},{{\text{HPO}}_{4}}^{2-},{\text{H}}_{2}{\text{PO}}_{4}{}^{-},\text{Urea},{\text{NH}}_{3},{{\text{NH}}_{4}}^{+},{\text{H}}^{+},{{\text{HCO}}_{2}}^{+},{\text{H}}_{2}{\text{CO}}_{2},\text{Gluc}\}, \end{array}$$ (3)

which is divided into non-reacting (*NR*) and reacting (*R*) solutes

$$\begin{array}{c} S_{NR}=\{{\text{Na}}^{+},{\text{K}}^{+},{\text{Cl}}^{-},\text{Urea},\text{Gluc}\} \end{array}$$ (4)

$$\begin{array}{c} S_{R}=\{{{\text{HCO}}_{3}}^{-},{\text{CO}}_{2},{\text{H}}_{2}{\text{CO}}_{3},{{\text{HPO}}_{4}}^{2-},{\text{H}}_{2}{\text{PO}}_{4}{}^{-},{\text{NH}}_{3},{{\text{NH}}_{4}}^{+},{\text{H}}^{+},{{\text{HCO}}_{2}}^{+},{\text{H}}_{2}{\text{CO}}_{2}\} \end{array}$$ (5)

In the W-PCT-E model, the system of state equations represents two different compartments: the cell and the lateral intercellular spaces. To formulate the mass conservation equations within each compartment, the net generation of each species *S*_*α*_(*i*) is defined as an intermediate variable within the compartment [11]. The generation of **multiple reacting** solutes is the sum of the net exchange of the flux plus the accumulation of that solute in each compartment, that is

$$\begin{array}{ccc} S_{I}\left(i\right)=J_{IE}\left(i\right)+J_{IS}\left(i\right)-J_{MI}\left(i\right)+\frac{d}{dt}\left[V_{I}C_{I}\left(i\right)\right] & & i\in S, \end{array}$$ (6)

$$\begin{array}{ccc} S_{E}\left(i\right)=J_{ES}\left(i\right)-J_{ME}\left(i\right)-J_{IE}\left(i\right)+\frac{d}{dt}\left[V_{E}C_{E}\left(i\right)\right] & & i\in S, \end{array}$$ (7)

where *S*_*I*_(*i*) and *S*_*E*_(*i*) indicate the generation for solute i∈S in the cell space and lateral interspace compartments, respectively. Within the epithelium, the flux of solute *i* across the membrane *αβ* is denoted as *J*_*αβ*_(*i*) [mmol. s^−1^. cm^−2^], αβ∈M and *V*_*α*_ is the compartment volume per unit surface area [cm^3^/*cm*^2^. epithelium], α∈C. The principle of mass conservation relating water fluxes and volume change for different compartments reads

$$\begin{array}{c} S_{I}\left(v\right)=J_{vIE}+J_{vIS}-J_{vMI}+\frac{d}{dt}\left[V_{I}\right], \end{array}$$ (8)

$$\begin{array}{c} S_{E}\left(v\right)=J_{vES}-J_{vME}-J_{vIE}+\frac{d}{dt}\left[V_{E}\right], \end{array}$$ (9)

where *J*_*vαβ*_ [ml. s^−1^. cm^−2^] is denoted as the transmembrane volume flux. It is important to mention that for nonreacting solutes we have

$$\begin{array}{ccc} S_{\alpha }\left(i\right)=0 & & \alpha \in C\;i\in S_{NR}, \end{array}$$ (10)

$$\begin{array}{ccc} S_{\alpha }\left(v\right)=0 & & \alpha \in C. \end{array}$$ (11)

The mass conservation then defines the change of the concentration of the i∈S species in the intracellular solution as the transport of solute *i* into and out of the cell through the apical and basolateral membrane. This is a direct transport of solutes through the membrane, and a contribution from convective transport due to the flow of water through the membranes. For such a nonreacting solute, the combination of (6) and (8) yields

$$\begin{array}{c} V_{I}\frac{dC_{I}\left(i\right)}{dt}=J_{MI}\left(i\right)-J_{IS}\left(i\right)-J_{IE}\left(i\right)-C_{I}\left(i\right)\left(J_{vMI}-J_{vIS}-J_{vIE}\right)\;i\in S \end{array}$$ (12)

This equation holds for each solute i∈S being considered in a particular instantiation of the model. If required for a particular model, similar equations can be introduced for the solute concentrations in the mucosal and/or serosal solutions.

Consequently, from (8), the conservation of cellular water yields the equation below, with the rate of change of cell volume, *V*_*I*_, defined as

$$\begin{array}{c} \frac{dV_{I}}{dt}=J_{vMI}-J_{vIS}-J_{vIE}, \end{array}$$ (13)

where each of the total membrane water fluxes, *J*_*vαβ*_, αβ∈M, is scaled (multiplied) by its respective membrane area to take into account the averaged behaviour of the representative membrane. For more detailed information of all various fluxes across the membrane, see Section IV.

To calculate the concentration of the cellular buffer pairs, the following expression is employed [11]

$$\begin{array}{c} \left(C_{{\text{Buf}}^{-}}+C_{\text{HBuf}}\right)V_{I}=C_{\text{TBuf}}V_{0I}. \end{array}$$ (14)

where $C_{{\text{Buf}}^{-}}$ and *C*_HBuf_ are cell buffer and protonated cell buffer concentration, respectively. *V*_*I*_ and *V*_0*I*_ indicate the cell volume and cell volume reference, respectively. To add more constraints on the proton cellular concentration, and to generate the paired equation, we have the following equilibrium equations

$$\begin{array}{c} pH=pK_{{{\text{HPO}}_{4}}^{2-}}+\;{\text{log}}_{10}(\frac{C_{{{\text{HPO}}_{4}}^{2-}}}{C_{{\text{H}}_{2}{\text{PO}}_{4}{}^{-}}}), \end{array}$$ (15)

$$\begin{array}{c} pH=pK_{{\text{Buf}}^{-}}+\;{\text{log}}_{10}(\frac{C_{{\text{Buf}}^{-}}}{C_{\text{HBuf}}}), \end{array}$$ (16)

where $pK_{{{\text{HPO}}_{4}}^{2-}}$ and $pK_{{\text{Buf}}^{-}}$ are the equilibrium constants for the phosphate and cell buffer pairs. Considering that the concentration of the *H*^+^ remains constant for all buffer pairs in the model, expressions (15) and (16) are combined to give

$$\begin{array}{c} pK_{{\text{Buf}}^{-}}+\;{\text{log}}_{10}(\frac{C_{{\text{Buf}}^{-}}}{C_{\text{HBuf}}})=pK_{{{\text{HPO}}_{4}}^{2-}}+\;{\text{log}}_{10}(\frac{C_{{{\text{HPO}}_{4}}^{2-}}}{C_{{\text{H}}_{2}{\text{PO}}_{4}{}^{-}}}). \end{array}$$ (17)

To include cellular proton concentration in the conservation of proton mass, *S*_*I*_(H^+^), the following modification is applied to the Eq (6)

$$\begin{array}{c} S_{I}\left({\text{H}}^{+}\right)=J_{IE}\left({\text{H}}^{+}\right)+J_{IS}\left({\text{H}}^{+}\right)-J_{MI}\left({\text{H}}^{+}\right)+\frac{d}{dt}\left[V_{I}C_{I}\left({\text{H}}^{+}\right)\right]-\frac{d}{dt}\left[V_{I}C_{{\text{Buf}}^{-}}\right], \end{array}$$ (18)

which can equivalently be written in the following form

$$\begin{array}{c} S_{I}\left({\text{H}}^{+}\right)=J_{IE}\left({\text{H}}^{+}\right)+J_{IS}\left({\text{H}}^{+}\right)-J_{MI}\left({\text{H}}^{+}\right)+\frac{d}{dt}\left[V_{I}C_{I}\left({\text{H}}^{+}\right)\right]+\frac{d}{dt}\left[V_{I}C_{\text{HBuf}}\right]. \end{array}$$ (19)

### ii. Buffer pairs and pH equilibrium

The W-PCT-E model defines different types of buffer pairs, the mass conservation principle for the phosphate and formate buffer pairs takes the form

$$\begin{array}{ccc} S_{\alpha }\left({\text{HPO}}_{4}{}^{2-}\right)+S_{\alpha }\left({\text{H}}_{2}{\text{PO}}_{4}{}^{-}\right)=0 & & \alpha \in \{E,I\}, \end{array}$$ (20)

$$\begin{array}{ccc} S_{\alpha }\left({{\text{HCO}}_{2}}^{+}\right)+S_{\alpha }\left({\text{H}}_{2}{\text{CO}}_{2}\right)=0 & & \alpha \in \{E,I\}. \end{array}$$ (21)

Similar equations apply to the ammonia pair within the lateral interspace and cell [11], that is

$$\begin{array}{c} S_{\alpha }\left({\text{NH}}_{3}\right)+S_{\alpha }\left({{\text{NH}}_{4}}^{+}\right)=0\;\;\;\alpha \in \left\{E,I\right\}, \end{array}$$ (22)

For an impermeant cytosolic buffer, Buf^−^, it is

$$\begin{array}{c} S_{I}\left(\text{Buf}\right)+S_{I}\left(\text{HBuf}\right)=0. \end{array}$$ (23)

All buffer species are assumed to be at chemical equilibrium. Within each compartment, there are four additional pH equilibrium relations, corresponding to the four buffer pairs. The algebraic relations of the model include the pH equilibria of four buffer pairs,

$$\begin{array}{c} pH=pK+\;{\text{log}}_{10}(\frac{{\text{Base}}^{-}}{\text{HBase}}). \end{array}$$ (24)

The collection of chosen buffer pairs and even the definition of mass conservation equation can be different over various studies depending on the specific focus of the study. As an example of the variability in these equations, one can compare the presentation of the mass conservation equation for NH_3_:NH_4_^+^ buffer pairs within a cell. In Weinstein’s reports from 1992 and 2009, see [11, 21], the authors introduced Eq (22) to define the mass conservation equation for these buffer pairs within a cell, while in [12] they used the following equation

$$\begin{array}{c} S_{I}\left({\text{NH}}_{3}\right)+S_{I}\left({{\text{NH}}_{4}}^{+}\right)=Q_{I}\left({{\text{NH}}_{4}}^{+}\right), \end{array}$$ (25)

where *Q*_*I*_(NH_4_^+^) is defined as an ammoniagenesis factor, see [12]. According to [11, 21], conservation of charge among the buffer reactions requires that

$$\begin{array}{c} S_{\alpha }\left({\text{H}}^{+}\right)+S_{\alpha }\left({{\text{NH}}_{4}}^{+}\right)=S_{\alpha }\left({{\text{HCO}}_{3}}^{-}\right)+S_{\alpha }\left({\text{HPO}}_{4}{}^{2-}\right)+S_{\alpha }\left({{\text{HCO}}_{2}}^{+}\right)\;\;\;\;\;\alpha \in \left\{I,E\right\}. \end{array}$$ (26)

We should mention that in [12], the authors employed a modified version of expression (26) as the requirement of the charge conservation among the cellular buffer reaction in the following form

$$\begin{array}{c} S_{I}\left({\text{H}}^{+}\right)+S_{I}\left({{\text{NH}}_{4}}^{+}\right)=S_{I}\left({{\text{HCO}}_{3}}^{-}\right)+S_{I}\left({\text{HPO}}_{4}{}^{2-}\right)+S_{I}\left({{\text{HCO}}_{2}}^{+}\right)+S_{I}\left({\text{Buf}}^{-}\right). \end{array}$$ (27)

In turn, the charge conservation for the lateral interspace (E) buffer reaction stays unchanged, as given by (26).

Although peritubular PCO_2_ is specified, the CO_2_ concentrations in cell and interspace are model variables. The mass conservation for HCO_3_^−^, H_2_CO_3_, CO_2_ is expressed as below

$$\begin{array}{cccc} & S_{\alpha }\left({\text{H}}_{2}{\text{Co}}_{3}\right)+S_{\alpha }\left({{\text{HCO}}_{3}}^{-}\right)=V_{\alpha }\left[k_{h}C_{\alpha }{\left({\text{CO}}_{2}\right)}_{\alpha }-k_{d}C_{\alpha }\left({\text{H}}_{2}{\text{Co}}_{3}\right)\right] & & \alpha \in \{E,I\}, \end{array}$$ (28)

$$\begin{array}{cccc} & S_{\alpha }\left({\text{H}}_{2}{\text{Co}}_{3}\right)+S_{\alpha }\left({{\text{HCO}}_{3}}^{-}\right)+S_{\alpha }\left({\text{CO}}_{2}\right)=0 & & \alpha \in \{E,I\}, \end{array}$$ (29)

where *k*_*h*_, *k*_*d*_ are the hydration and dehydration rates for CO_2_, respectively.

The buffering described above and implemented in our unified W-PCT-E model provides a flexible and modular method to explore various features of the system. This covers the broadest range of buffering included in the Weinstein models while enabling replication of specific instances where different approaches are used. For example [12], in which the impermeant buffer conservation equation was omitted, resulting in the expression (22) being replaced by (25) and for the expression (26) is replaced by (27).

### iii. Electroneutrality constraints

When considering the movement of charged solutes, with valence *Z*_*i*_, i∈S, the proposed system of equations is not sufficient to guarantee that the cell and interspace remain electrically neutral. An electroneutrality relation for the cell compartment is expressed through the following balance equation

$$\begin{array}{c} \sum_{i\in S}Z_{i}C_{I}\left(i\right)+Z_{I,\text{Imp}}C_{I,\text{Imp}}-C_{{\text{Buf}}^{-}}=0, \end{array}$$ (30)

where *C*_Imp_ and $C_{{\text{Buf}}^{-}}$ denote the concentration of cell impermeant solute and cell unprotonated buffer, *Z*_*I*,Imp_ is the cell impermeant valence, and *Z*_*i*_ is the valence of species *i*, i∈S. The law of electroneutrality for the interspace is defined as follows

$$\begin{array}{c} \sum_{i\in S}Z_{i}C_{E}\left(i\right)=0, \end{array}$$ (31)

and for all of the buffer reactions, there is conservation of protons, which implies that the following is verified

$$\begin{array}{c} \sum_{i\in S}Z_{i}S_{\alpha }\left(i\right)=0\;\;\;\;\alpha \in C. \end{array}$$ (32)

The electroneutrality condition is effectively prescribed by considering that the net charge fluxes into and out of the epithelium are the same. The membrane charge fluxes can be represented as electrical currents using the following relationships

$$\begin{array}{c} I_{In}=I_{MI}+I_{ME}=F(\sum_{i\in S}Z_{i}J_{MI}\left(i\right)+\sum_{i\in S}Z_{i}J_{ME}\left(i\right)), \end{array}$$ (33)

$$\begin{array}{c} I_{Out}=I_{IS}+I_{ES}=F(\sum_{i\in S}Z_{i}J_{IS}\left(i\right)+\sum_{i\in S}Z_{i}J_{ES}\left(i\right)), \end{array}$$ (34)

where *F* is Faraday’s constant. Balancing the flow of charge into and out of the epithelium therefore results in

$$\begin{array}{c} I_{\text{Out}}=I_{\text{In}}. \end{array}$$ (35)

which must hold true at all times. In the current work, according to [12], expression (3) is only considered for the balance of charge transfer across the membranes such that *I*_In_ = 0. Eqs (26)–(34) are a collection of different electroneutrality constraints that were introduced in different studies (e.g., [11, 12, 21]). However, it is important to note that not all these equations were utilised in all different studies. Instead, they were selectively chosen based on the context of each study.

## IV. Model specialisation

The basic principles of mass conservation, pH equilibrium of buffer species, and maintenance of electroneutrality described above apply to epithelial transport in general. To instantiate the general model into a mathematical model for a specific epithelium, all that remains is to define the actual membrane solute and water fluxes of interest to create the specialised model.

### i. Water fluxes

With respect to water fluxes, the volume conservation equations for lateral interspace and cell are considered to compute the lateral interspace hydrostatic pressure, and cell volume. Across each cell membrane, the transmembrane water fluxes are proportional to the hydrostatic, oncotic, and osmotic driving forces

$$\begin{array}{c} J_{v\alpha \beta }=L_{p\alpha \beta }A_{\alpha \beta }\left(P_{\alpha }-P_{\beta }\right)-L_{p\alpha \beta }A_{\alpha \beta }\left(\pi_{\alpha }-\pi_{\beta }\right)+L_{p\alpha \beta }A_{\alpha \beta }RT(\sum_{i\in S}\sigma_{\alpha \beta }\left(i\right)(C_{\alpha }\left(i\right)-C_{\beta }\left(i\right))\;\;\;\;\;\alpha \beta \in M, \end{array}$$ (36)

where *P*_*α*_ and *π*_*α*_ are the hydrostatic and oncotic pressures within compartment α∈C, *L*_*pαβ*_ is the membrane water permeability and *σ*_*αβ*_(*i*) is the reflection coefficient of membrane αβ∈M to solute i∈S, and *R* and *T* are the gas constant and absolute temperature, respectively. It is important to mention that all *L*_*pαβ*_ constants which are represented in the implementation of this model (see the Python code) are multiplied by *R* and *T*. The reflection coefficients, *σ*_*αβ*_(*i*), stay identical in most of Weinstein’s body of work, see Table 1 in S1 File. In turn, there are some variations in the model parameters (such as coupled transporter coefficients, cell membrane water permeability, and cell membrane solute permeability) across the different works.

### ii. Convective solute fluxes

In the model proposed in [11], it is assumed that there are convective fluxes for all intraepithelial solutes. Taking into account the definition of the water fluxes, see (36), the convective fluxes are defined as follows [12]

$$\begin{array}{c} J_{\alpha \beta }^{C}\left(i\right)=J_{v\alpha \beta }\left(1-\sigma_{\alpha \beta }\left(i\right)\right){\bar{C}}_{\alpha \beta }\left(i\right)\;\;\;\;\alpha \beta \in M\;\;\;i\in S, \end{array}$$ (37)

where ${\bar{C}}_{\alpha \beta }\left(i\right)$ is the logarithmic mean membrane solute concentration described by the expression

$$\begin{array}{c} {\bar{C}}_{\alpha \beta }\left(i\right)=\frac{C_{\alpha }\left(i\right)-C_{\beta }\left(i\right)}{\text{log}C_{\alpha }\left(i\right)-\text{log}C_{\beta }\left(i\right)}\;\;\;\;\;\alpha \beta \in M\;\;\;i\in S. \end{array}$$ (38)

Studying the reflection coefficient values *σ*_*αβ*_(*i*) (defined as membrane-solute properties), one can see that the reflection coefficient is mostly one in lumen-cell (MI), cell-lateral (IE) and cell basal membrane (IS). The most effective membrane to produce the convective fluxes is the interspace basement membrane (ES) with the reflection coefficient mostly zero, and then in the second place it is the tight junction (ME).

### iii. Passive solute fluxes

In the W-PCT-E model, passive solute fluxes across all membranes are assumed to occur by electrodiffusion and to conform to the Goldman-Hodgkin-Katz constant-field flux equation [22]. Passive solute fluxes of the species i∈S across the membrane αβ∈M are given by

$$\begin{array}{c} J_{\alpha \beta }^{P}\left(i\right)=h_{\alpha \beta }\left(i\right)A_{\alpha \beta }\zeta_{\alpha \beta }\left(i\right)(\frac{C_{\alpha }\left(i\right)-C_{\beta }\left(i\right)\text{exp}\left(-\zeta_{\alpha \beta }\right)}{1-\text{exp}(-\zeta_{\alpha \beta })})\;\;\;\;\;\alpha \beta \in M\;\;\;i\in S, \end{array}$$ (39)

where, for solute i∈S and for membrane αβ∈M, *h*_*αβ*_(*i*) [cm.*s*^−1^] is the permeability and *ζ*_*αβ*_(*i*) is a normalised electrical potential difference (dimensionless), defined by

$$\begin{array}{c} \zeta_{\alpha \beta }\left(i\right)=\frac{Z_{i}F}{RT}\left(\psi_{\alpha }-\psi_{\beta }\right)\;\;\;\;\;\alpha \beta \in M\;\;\;i\in S. \end{array}$$ (40)

where *ψ*_*α*_ and *ψ*_*β*_ are electrical potentials within compartments *α* and *β*, respectively. *F* is Faraday’s constant, *R* and *T* are the gas constant and absolute temperature, sequentially. Weinstein et al. did not hold on to one solid definition for the permeability, in some cases permeability was multiplied by the area of the corresponding membrane *h*_*αβ*_(*i*)*A*_*αβ*_[10^−5^ cm^3^.s^−1^.cm^−2^. epithelium] as an example see Weinstein et al. [12]. For the uncoupled permeation of neutral solutes across membranes, the Fick law is utilised

$$\begin{array}{c} J_{\alpha \beta }^{P}\left(i\right)=h_{\alpha \beta }\left(i\right)A_{\alpha \beta }\left(C_{\alpha }\left(i\right)-C_{\beta }\left(i\right)\right)\;\;\;\;\;\alpha \beta \in M\;\;\;i\in S. \end{array}$$ (41)

### iv. Coupled solute fluxes

Coupled solute fluxes in the W-PCT-E model include three different categories of transporters: simple cotransporters, simple exchangers, and complex exchangers. All coupled solute transporters in this model have been represented according to linear nonequilibrium thermodynamics, so that the solute permeation rates are proportional to the electrochemical driving force of the aggregate species, with a single permeation coefficient. Simple cotransporters consist of peritubular K^+^—Cl^−^, luminal Na^+^—Gluc and Na^+^—H_2_PO_4_^−^, which are in the form of the following equations

$$\begin{array}{c} {}[\begin{array}{c} J_{IS}^{E}\left({\text{K}}^{+}\right)\\ J_{IS}^{E}\left({\text{Cl}}^{-}\right) \end{array}]=L_{\left({\text{K}}^{+},{\text{Cl}}^{-}\right)}[\begin{array}{cc} 1 & 1\\ 1 & 1 \end{array}][\begin{array}{c} {\bar{\mu }}_{IS}\left({\text{K}}^{+}\right)\\ {\bar{\mu }}_{IS}\left({\text{Cl}}^{-}\right) \end{array}], \end{array}$$ (42)

$$\begin{array}{c} {}[\begin{array}{c} J_{MI}^{E}\left({\text{Na}}^{+}\right)\\ J_{MI}^{E}\left(\text{Gluc}\right) \end{array}]=L_{\left({\text{Na}}^{+},\text{Gluc}\right)}[\begin{array}{cc} 1 & 1\\ 1 & 1 \end{array}][\begin{array}{c} {\bar{\mu }}_{MI}\left({\text{Na}}^{+}\right)\\ {\bar{\mu }}_{MI}\left(\text{Gluc}\right) \end{array}], \end{array}$$ (43)

$$\begin{array}{c} {}[\begin{array}{c} J_{MI}^{E}\left({\text{Na}}^{+}\right)\\ J_{MI}^{E}\left({\text{H}}_{2}{\text{PO}}_{4}{}^{-}\right) \end{array}]=L_{\left({\text{Na}}^{+},{\text{H}}_{2}{\text{PO}}_{4}{}^{-}\right)}[\begin{array}{cc} 1 & 1\\ 1 & 1 \end{array}][\begin{array}{c} {\bar{\mu }}_{MI}\left({\text{Na}}^{+}\right)\\ {\bar{\mu }}_{MI}\left({\text{H}}_{2}{\text{PO}}_{4}{}^{-}\right) \end{array}]. \end{array}$$ (44)

It is important to mention that all transporters within cell-basal (IS) membrane are also considered for the cell-lateral (IE) membrane.

In the equations above, the fluxes of two different species across the cotransporter are equal (1: 1 stoichiometry).

Simple exchangers such as Na^+^/H^+^, Na^+^/NH_4_^+^, Cl^−^/HCO_2_^−^, and Cl^−^/HCO_3_^−^ are located at the lumen-cell (MI) membrane, and represented by the equations below

$$\begin{array}{c} {}[\begin{array}{c} J_{MI}^{E}\left({\text{Na}}^{+}\right)\\ J_{MI}^{E}\left({\text{H}}^{+}\right) \end{array}]=L_{\left({\text{Na}}^{+},{\text{H}}^{+}\right)}[\begin{array}{cc} 1 & -1\\ -1 & 1 \end{array}][\begin{array}{c} {\bar{\mu }}_{MI}\left({\text{Na}}^{+}\right)\\ {\bar{\mu }}_{MI}\left({\text{H}}^{+}\right) \end{array}], \end{array}$$ (45)

$$\begin{array}{c} {}[\begin{array}{c} J_{MI}^{E}\left({\text{Na}}^{+}\right)\\ J_{MI}^{E}\left({{\text{NH}}_{4}}^{+}\right) \end{array}]=L_{\left({\text{Na}}^{+},{{\text{NH}}_{4}}^{+}\right)}[\begin{array}{cc} 1 & -1\\ -1 & 1 \end{array}][\begin{array}{c} {\bar{\mu }}_{MI}\left({\text{Na}}^{+}\right)\\ {\bar{\mu }}_{MI}\left({{\text{NH}}_{4}}^{+}\right) \end{array}], \end{array}$$ (46)

$$\begin{array}{c} {}[\begin{array}{c} J_{MI}^{E}\left({\text{Cl}}^{-}\right)\\ J_{MI}^{E}\left({{\text{HCO}}_{2}}^{+}\right) \end{array}]=L_{\left({\text{Cl}}^{-},{{\text{HCO}}_{2}}^{+}\right)}[\begin{array}{cc} 1 & -1\\ -1 & 1 \end{array}][\begin{array}{c} {\bar{\mu }}_{MI}\left({\text{Cl}}^{-}\right)\\ {\bar{\mu }}_{MI}\left({{\text{HCO}}_{2}}^{+}\right) \end{array}], \end{array}$$ (47)

$$\begin{array}{c} {}[\begin{array}{c} J_{MI}^{E}\left({\text{Cl}}^{-}\right)\\ J_{MI}^{E}\left({{\text{HCO}}_{3}}^{-}\right) \end{array}]=L_{\left({\text{Cl}}^{-},{{\text{HCO}}_{3}}^{-}\right)}[\begin{array}{cc} 1 & -1\\ -1 & 1 \end{array}][\begin{array}{c} {\bar{\mu }}_{MI}\left({\text{Cl}}^{-}\right)\\ {\bar{\mu }}_{MI}\left({{\text{HCO}}_{3}}^{-}\right) \end{array}]. \end{array}$$ (48)

In [11], the authors introduced the NHE3 exchanger in the luminal membrane through Eqs (45) and (46). However, the NHE3 exchanger has been developed through the kinetic model proposed by Weinstein et al. in 1995 [20]. In the current work, the NHE3 exchanger is modelled by employing the mathematical system introduced in [20]. There are also two more complex transporters at the peritubular membrane: Na^+^—HCO_3_^−^ and Na^+^—2HCO_3_^−^/Cl^−^ which are defined by the following equations (see [11])

$$\begin{array}{c} {}[\begin{array}{c} J_{IS}^{E}\left({\text{Na}}^{+}\right)\\ J_{IS}^{E}\left({{\text{HCO}}_{3}}^{-}\right) \end{array}]=L_{\left({\text{Na}}^{+},{{\text{HCO}}_{3}}^{-}\right)}[\begin{array}{cc} 1 & 3\\ 3 & 9 \end{array}][\begin{array}{c} {\bar{\mu }}_{IS}\left({\text{Na}}^{+}\right)\\ {\bar{\mu }}_{IS}\left({{\text{HCO}}_{3}}^{-}\right) \end{array}], \end{array}$$ (49)

$$\begin{array}{c} {}[\begin{array}{c} J_{IS}^{E}\left({\text{Na}}^{+}\right)\\ J_{IS}^{E}\left({\text{Cl}}^{-}\right)\\ J_{IS}^{E}\left({{\text{HCO}}_{3}}^{-}\right) \end{array}]=L_{\left({\text{Na}}^{+},{\text{Cl}}^{-},{{\text{HCO}}_{3}}^{-}\right)}[\begin{array}{ccc} 1 & -1 & 2\\ -1 & 1 & -2\\ 2 & -2 & 4 \end{array}][\begin{array}{c} {\bar{\mu }}_{IS}\left({\text{Na}}^{+}\right)\\ {\bar{\mu }}_{IS}\left({\text{Cl}}^{-}\right)\\ {\bar{\mu }}_{IS}\left({{\text{HCO}}_{3}}^{-}\right) \end{array}]. \end{array}$$ (50)

In the expressions above, *L*_(*i*,*j*)_ is the transporter coupling coefficient, a single proportionality constant which specifies the relative activity of the transporters. In turn, ${\bar{\mu }}_{\alpha \beta }\left(i\right)$ is the electrochemical potential difference of species *i* across all membranes

$$\begin{array}{c} {\bar{\mu }}_{\alpha \beta }\left(i\right)=RT\;\text{ln}\;\frac{C_{\alpha }\left(i\right)}{C_{\beta }\left(i\right)}+Z_{i}F\psi_{\alpha \beta }. \end{array}$$ (51)

Here, we also define the following quantity

$$\begin{array}{c} {\bar{\mu }}_{\alpha }=RT\;\text{ln}\;C_{\alpha }\left(i\right)+Z_{i}F\psi_{\alpha }. \end{array}$$ (52)

It is important to mention that all permeability coefficients, *L*_(*i*,*j*)_, which are represented in Table 1, from [11], are scaled (multiplied) by their respective membrane area to take into account the effective behaviour of the representative membrane.

### v. Active solute fluxes

In the W-PCT-E model, there are two ATPases, the apical membrane H^+^—ATPase and a peritubular Na^+^/K^+^—ATPase. To model the H^+^—ATPase, an expression of the following form is utilised

$$\begin{array}{c} J_{MI}^{A}\left({\text{H}}^{+}\right)=\frac{{\left[J_{MI}\left({\text{H}}^{+}\right)\right]}_{\text{max}}}{1.0+\;\text{exp}\;[\epsilon_{MI}\left({\bar{\mu }}_{MI}\left({\text{H}}^{+}\right)-{\bar{\mu }}_{0}\right)]}. \end{array}$$ (53)

**Fig 3:**
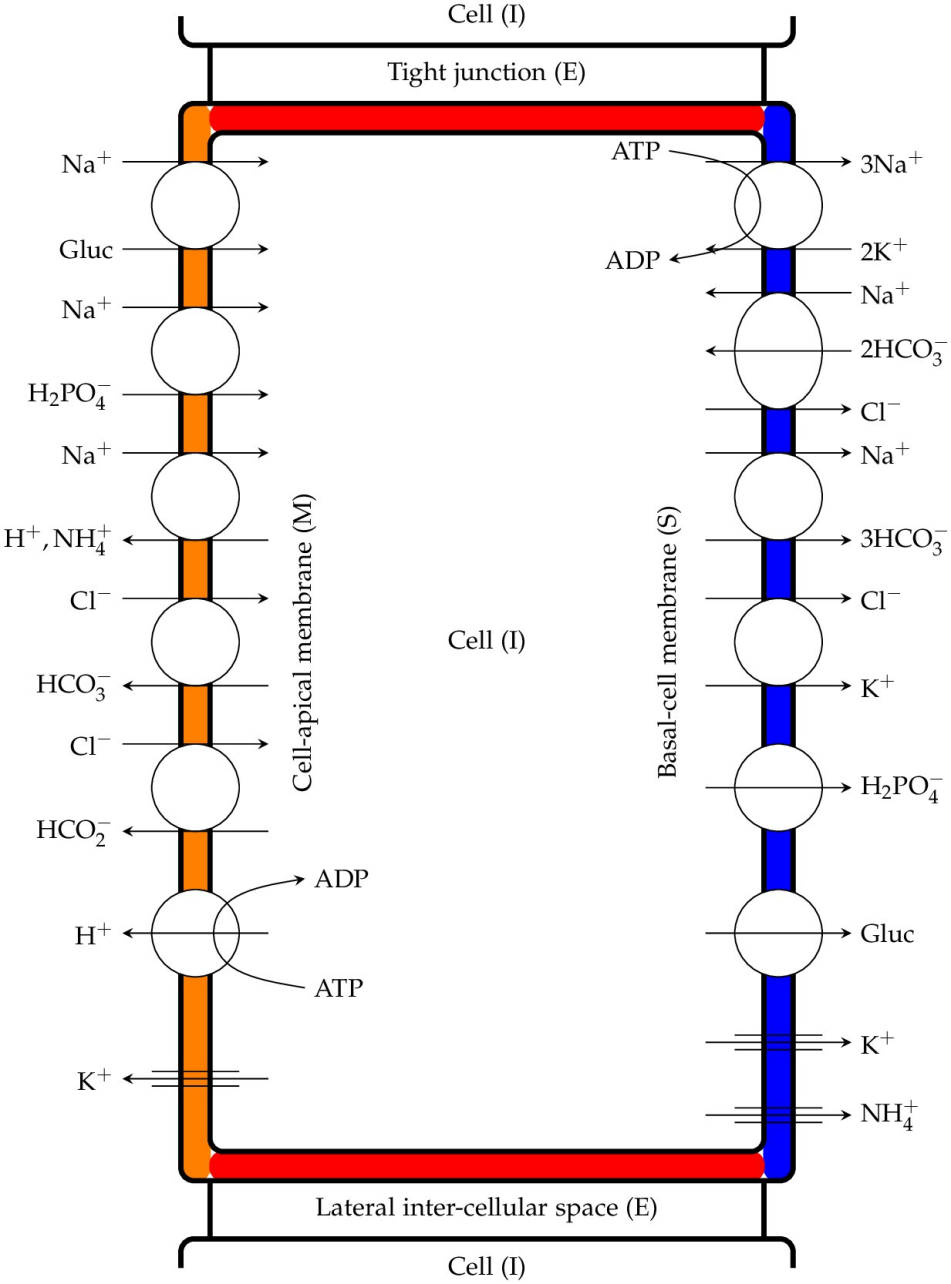
Proximal tubule cells showing coupled transport pathways and some ion channels within the luminal and peritubular cell membranes. It is essential to mention that peritubular cell and cell-lateral membranes share the same feature regarding their transport pathways, even though it has not been shown in this diagram.

Note that the rate of proton pumping varies as a function of the transmembrane electrochemical potential difference. Here, [*J*_*MI*_(H^+^)]_max_ is a maximal rate of transport, and *ϵ*_*MI*_ is a steepness coefficient. The Na^+^/K^+^ − ATPase exchanges three cytosolic Na^+^ ions for two peritubular cations, K^+^ or NH_4_^+^, in a way that competes for binding. The following expressions represent all three different fluxes due to the Na^+^/K^+^ − ATPase activities

$$\begin{array}{c} J_{IS}^{A}\left({\text{Na}}^{+}\right)={\left[J_{IS}^{A}\left({\text{Na}}^{+}\right)\right]}_{\text{max}}A_{IS}[\frac{C_{I}\left({\text{Na}}^{+}\right)}{C_{I}\left({\text{Na}}^{+}\right)+K_{\text{Na}+}}{]}^{3}[\frac{C_{S}\left({\text{K}}^{+}\right)+C_{S}\left({{\text{NH}}_{4}}^{+}\right)}{C_{S}\left({\text{K}}^{+}\right)+C_{S}\left({{\text{NH}}_{4}}^{+}\right)+K_{{\text{K}}^{+}}}{]}^{2}, \end{array}$$ (54)

$$J_{IS}^{A}\left({\text{K}}^{+}\right)=-\frac{2}{3}J_{IS}^{A}\left({\text{Na}}^{+}\right)-J_{IS}^{A}\left({{\text{NH}}_{4}}^{+}\right),$$ (55)

$$\frac{J_{IS}^{A}\left({{\text{NH}}_{4}}^{+}\right)}{J_{IS}^{A}\left({\text{K}}^{+}\right)}=\frac{C_{S}\left({{\text{NH}}_{4}}^{+}\right)}{K_{{{\text{NH}}_{4}}^{+}}}\frac{K_{{\text{K}}^{+}}}{C_{S}\left({\text{K}}^{+}\right)},$$ (56)

$$K_{\text{Na}+}=0.2\cdot 10^{-3}[1.0+\frac{C_{I}\left({\text{K}}^{+}\right)}{8.33\cdot 10^{-3}}],$$ (57)

$$K_{{\text{K}}^{+}}=K_{{{\text{NH}}_{4}}^{+}}=0.1\cdot 10^{-3}[1.0+\frac{C_{I}\left({\text{Na}}^{+}\right)}{18.5\cdot 10^{-3}}],$$ (58)

in which $K_{{\text{Na}}^{+}}$, the half-maximal of Na^+^ concentration, which scales linearly with the cellular concentration of K^+^; $K_{{\text{K}}^{+}}$, which is the half-maximal of K^+^ concentration, rises linearly with the external concentration of K^+^. The pump flux of K^+^ plus NH_4_^+^ reflects the 3:2 stoichiometry. Similar expressions are considered for active transport at the cell-lateral membrane (IE), denoted by $J_{IE}^{A}$. Fig 3 illustrates the proximal PCT cell featuring coupled transport pathways and ion channels within luminal and basolateral membranes, the coupled transport pathways within the cell-lateral are not included.

### vi. Total membrane solute fluxes

In summary, in the W-PCT-E model, the intraepithelial solute transport through membrane αβ∈M results from the contribution of convective flux $J_{\alpha \beta }^{C}$, passive flux $J_{\alpha \beta }^{P}$, electrodiffusive coupled flux $J_{\alpha \beta }^{EC}$, and/or metabolically driven flux $J_{\alpha \beta }^{A}$, that is

$$\begin{array}{c} J_{\alpha \beta }\left(i\right)=J_{\alpha \beta }^{C}\left(i\right)+J_{\alpha \beta }^{P}\left(i\right)+J_{\alpha \beta }^{EC}\left(i\right)+J_{\alpha \beta }^{A}\left(i\right)\;i\in S, \end{array}$$ (59)

where *J*_*αβ*_(*i*)[mmol.s.cm^−2^] is the total flux of *i* solute flow from the compartment *α* to the compartment *β* (through membrane *αβ*).

### vii. Model compliance parameters

In the W-PCT-E model, the cell is compliant in a manner that there is no hydrostatic pressure difference between the cell and lumen, therefore, we have *P*_*I*_ = *P*_*M*_. There is a substantial oncotic force within the cell, *π*_*I*_, that increases with decrements in the cell volume. Here, it is assumed that the total cell protein content *C*_*I*,Imp_ *V*_0*I*_ is fixed and that *π*_*I*_ is proportional to *C*_*I*,Imp_, for this reason *C*_*I*,Imp_ replaces *π*_*I*_ as one of the model unknowns (for more information see [11, 12])

$$\begin{array}{c} V_{I}=V_{I0}\frac{C_{I,\text{Imp}0}}{C_{I,\text{Imp}}}. \end{array}$$ (60)

The lateral interspace volume (*V*_*E*_) and its basement membrane area (*A*_*ES*_) are functions of interspace hydrostatic pressure, *P*_*E*_, that is

$$\begin{array}{c} A_{ES}=A_{ES0}\left(1+\nu_{A}\left(P_{E}-P_{I}\right)\right), \end{array}$$ (61)

$$\begin{array}{c} V_{E}=V_{E0}\left(1+\nu_{V}\left(P_{E}-P_{I}\right)\right), \end{array}$$ (62)

where *A*_*ES*0_ and *V*_*E*0_ are reference values for the outlet area and volume, *ν*_*A*_ and *ν*_*V*_ are the compliance parameters used to ensure that the suitable pressures are maintained in the system (see Table 1 in S1 File).

Table 1 in S1 File includes the model parameters and their definitions either in the current document or in the Python code. You can see the geometric parameters for all compartments in the tables reported below. As can be seen, the luminal and peritubular cell membranes have equal areas, i.e., 36 [cm^2^/cm^2^. epithelium]. The lateral interspace is compliant and distends with transport-associated increments in interspace pressure. In this model, the tight junction properties are fixed and do not vary with transjunctional pressure differences.

## V. Model calculations

In this work, the W-PCT-E model is bathed on both luminal and peritubular sides by solutions of equal concentration. Baseline bath and lumen conditions are those reported in Table 1 in S1 File. The choices for the model parameters appear in Table 2 in S1 File. The geometric parameters are completely different from [11], *A*_*IE*_= 35 [cm^2^/cm^2^. epithelium], *A*_*IM*_ = 36 [cm^2^/cm^2^. epithelium]. As we mentioned earlier, the parameter values used in the current work are derived from information available in the literature and, in some cases, discovered in Weinstein’s original implementation [https://github.com/amweins/kidney-models-amw/blob/master/epithelia/pct/param.tem].

In the LIS interspace, the LIS basement membrane area and volume are compliant and distend with transport-associated increments in interspace pressure. However, the membrane areas in the cell are fixed and do not vary with transjunctional pressure differences. For a fixed quantity of cellular impermeant solutes, increasing the cell volume will decrease the impermeant concentration. The suitability of these parameters was not tested here. For the W-PCT-E simulations, the 35 nonlinear ordinary differential equations are solved using a finite difference numerical method for time discretisation along the Python solver “scipy.optimize.root”. Evaluation of the model involves integrating the mass conservation equations from an initial time to a final time using small time increments. The simulation time is chosen to ensure that a steady-state regime is reached. Here, we should highlight that the solver also provides the eigenvalues of the W-PCT-E model. Studying the eigenvalues demonstrates that the W-PCT-E model is strongly stable with negative real parts and zero imaginary parts (for more information, see [23]). To analyse the W-PCT-E model stability, we extracted the Jacobian matrices from the results provided by Python solver “scipy.optimize.root”. Then, we applied the built-in function “scipy.linalg.eig” to calculate the eigenvalues. Our stability results represent a stable system.

Here, there is a list of all the variables in the model: First, all variables appear in the lateral interspace compartment,

*V*_*E*_, *P*_*E*_, *C*_*E*_(Na^+^), *C*_*E*_(K^+^), *C*_*E*_(Cl^−^), *C*_*E*_(HCO_3_^−^), *C*_*E*_(H_2_CO_3_^−2^), *C*_*E*_(CO_2_), *C*_*E*_(HPO_4_^−^), *C*_*E*_(H_2_PO_4_^−2^), *C*_*E*_(urea), *C*_*E*_(NH_4_), *C*_*E*_(HCO_2_^−^), *C*_*E*_(H_2_CO_2_^−2^), *C*_*E*_(Gluc).

Then, all variables which appear in the cellular compartment,

*V*_*I*_, *C*_*I*,*Imp*_, *C*_*I*_(Na^+^), *C*_*I*_(K^+^), *C*_*I*_(Cl^−^), *C*_*I*_(HCO_3_^−^), *C*_*I*_(H_2_CO_3_^−2^), *C*_*I*_(CO_2_), *C*_*I*_(HPO_4_^−^), *C*_*I*_(H_2_PO_4_^−2^), *C*_*I*_(urea), *C*_*I*_(NH_4_), *C*_*I*_(HCO_2_^−^), *C*_*I*_(H_2_CO_2_^−2^), *C*_*I*_(Gluc), *C*_*I*_(Buf^−^), *C*_*I*_(HBuf), plus the only luminal variable which is the voltage inside the lumen *V*_*M*_. The Github link for the W-PCT-E Python code is https://github.com/iNephron/W-PCT-E.

## VI. Results

We investigated the validity of the WPCT-E model by designing several experiments; the analyses are performed over the steady-state solutions found from numerical simulations. To test the robustness of the W-PCT-E model, we investigated the sensitivity of steady-state solutions to different sets of initial conditions or time steps. Furthermore, we explored reproducibility by replicating some simulation experiments reported in [11, 12] using the W-PCT-E. We then investigated the sensitivity to salt intake or luminal salt concentration in the W-PCT-E model based on earlier work [24]. Structural analyses were performed by inhibiting key transporters in different membranes, such as the Na^+^/K^+^-ATPase in the peritubular membrane or SGLT, NHE3, and Na^+^-H_2_PO_4_ transporters in the apical membrane, and relating the predicted responses to observed biological phenomena.

### i. Model sensitivity analysis

A time step size no greater than Δ*t* = 0.1 s is required to ensure a converged numerical solution for epithelial transport models. In our simulations, we considered a time-step of Δ*t* = 0.1 s.

In exploring the sensitivity of our W-PCT-E model to the initial conditions, we found that as long as the initial conditions were within a reasonable physiological range that the steady-state solution was insensitive to the initial conditions (to check the initial conditions, see the https://github.com/iNephron/W-PCT-E). However, outside that range, the results of the model are highly variable. To ensure consistent behaviour across the range of simulation experiments presented here, we use the same initial conditions. To determine that initial state, we performed a simulation experiment whereby we disabled all active transporters and allowed our W-PCT-E model to reach a steady state using only the passive processes. This steady state was then used as the initial state for all subsequent simulation experiments. When a given simulation experiment requires the addition of active processes to the model, we follow a similar protocol starting from the passive steady state, introducing the active process(es), and allowing the system to reach a new steady state before introducing further perturbation. This ensures that the simulations then begin at a suitable initial state in which the predicted steady state solution is insensitive. Applying the above approach to define the initial conditions does not affect our simulation results or the behaviour of the model. Even when the cell volume substantially increases because of the process of defining the initial conditions, the procedure yields stable numerical simulations.

### ii. Model reproducibility

The present W-PCT-E model is built from a collection of mathematical representations reported in the literature. Our efforts have been to compile model parameters and equations from different scientific studies in which the components of PCT have been reported. Moreover, we provide the community with a freely available implementation to speed up research. In doing so, we aim to ensure that the behaviour of the W-PCT-E model reproduces that of the source model. Here, we provide exemplars exhibiting the flexibility and reproducibility of the W-PCT-E model.

#### Flow-dependent transport in the PCT

The mathematical model of the rat proximal tubule [12] was designed to include the calculation of microvillous torque and to incorporate torque-dependent solute transport in a compliant tubule. Here, we aim to reproduce some of the results reported in [12] by tuning the parameters according to [12] (see Tables 1–2 in that article). For those constant parameters or boundary conditions which were not defined in [12], we infer them from earlier works, specifically [10, 11]. Reproducing these simulation experiments, we found no significant discrepancies, as shown in Table 1, for steady-state solutions such as solute concentrations, luminal voltage, and luminal pressure.

**Table 1:** A comparison between the solutions obtained with the present W-PCT-E model and the model reported in [12]. Electrical potentials are in mV, pressures in mmHg and concentrations in mM.

Variables	Cell		Interspace	
	[12]	W-PCT-E	[12]	W-PCT-E
*V*	-55.6	-55.5	-0.01	-0.01
*P*	15	15	-23.1	-25.5
C(Na^+^)	19.6	19.6	140.3	139.2
C(K^+^)	138.1	137.4	4.6	4.6
C(Cl^−^)	16.3	15.9	112	111.2
C(HCO_3_^−^)	25.0	24.8	25.6	25.2
C(H_2_CO_3_^-2^)	4.3 *e*^−3^	4.3 *e*^−3^	4.3 *e*^−3^	4.3 *e*^−3^
C(CO_2_)	1.49	1.49	1.49	1.49
C(HPO_4_^−^)	8.5	8.3	2.9	2.9
C(H_2_PO_4_^-2^)	2.49	2.49	0.86	0.85
C(Urea)	4.9	4.9	4.91	4.86
C(NH_3_)	3.48 *e*^−3^	3.48 *e*^−3^	2.7 *e*^−3^	2.68 *e*^−3^
C(HCO_2_^−^)	0.52	0.42	0.77	0.79
C(H_2_CO_2_^-2^)	0.91 *e*^−3^	1.17 *e*^−3^	2.04 *e*^−3^	2.13 *e*^−3^
C(Gluc)	15	14.93	7.7	7.6
Impermeant	68.9	69.4	–	–

#### Chloride transport in the PCT

Here, we aim to reproduce some of the results from chloride transport in the proximal tubule [11] to explore the possible interactions between the individual transporter pathways and their contribution to overall chloride reabsorption in a proximal tubule. At the apical membrane, Cl^−^/HCO_2_^−^ and Cl^−^/HCO_3_^−^ exchangers are the main pathways for Cl^−^ entry, and across the peritubular membrane Cl^−^/2HCO_3_^−^-Na^+^ and Cl^−^-K^+^ for the exit of Cl^−^. At the tight junction, chloride fluxes are both diffusive and convective. We performed the same experiments as reported in [11] and observed that the W-PCT-E model (with parameters modified accordingly) predicted similar behaviour as [11].

**Fig 4:**
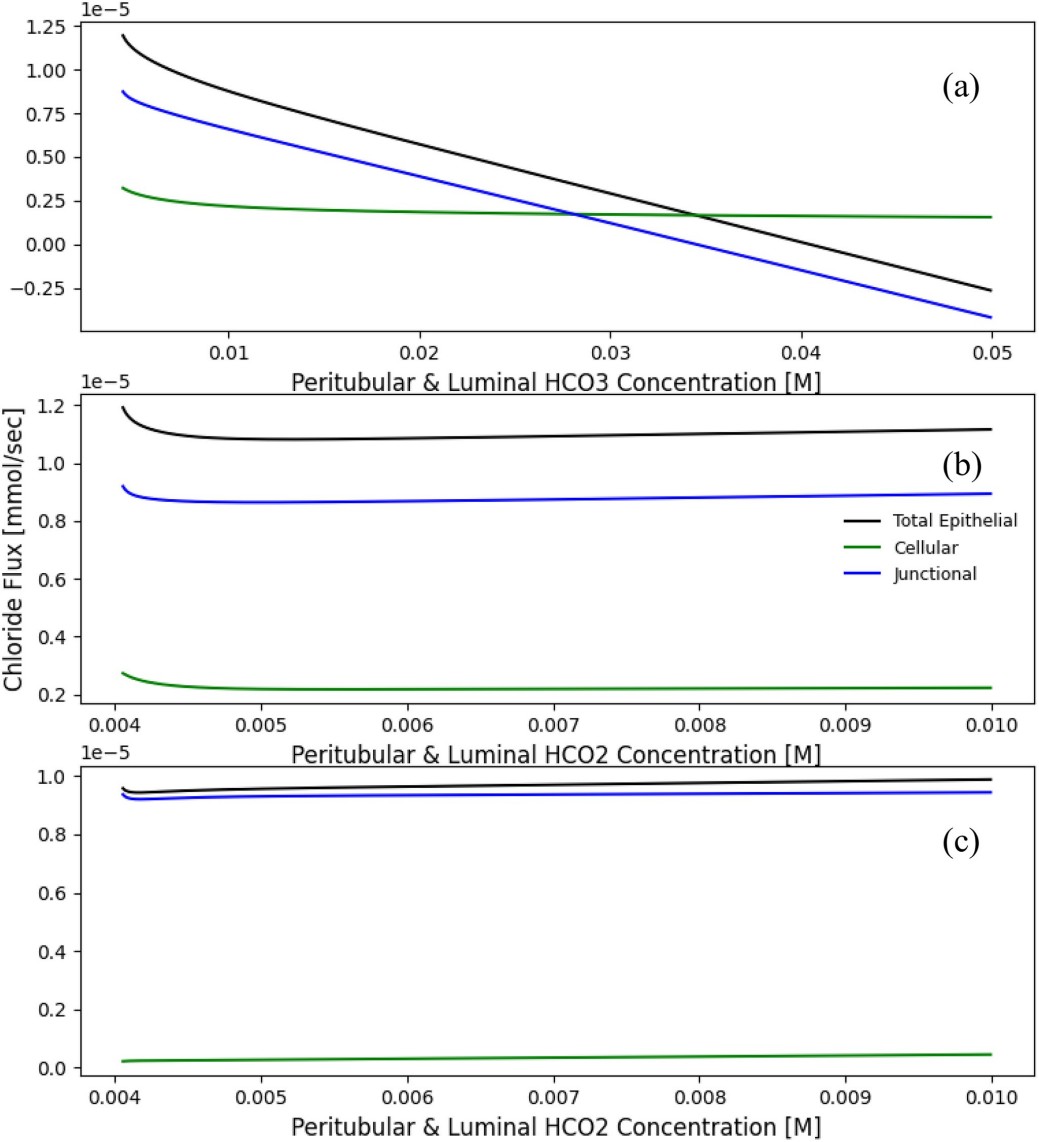
Total epithelial chloride fluxes for the W-PCT-E model. Panel (a) represents the effect of luminal HCO_3_^−^ concentration on Cl^−^ fluxes (c.f. Fig 9, [11]). Panel (b) represents the effect of ambient HCO_2_^−^ concentration on Cl^−^ fluxes (c.f. Fig 11, [11]). Luminal concentration for HCO_3_^−^ is considered to be as low as 0.004 M, while the peritubulur concentration for HCO_3_^−^ stays the same 0.024 M. Luminal and peritubular formate are varied simultaneously from 0.003 to 0.05. Panel (c) shows the effect of ambient HCO_2_^−^ concentration on Cl^−^ fluxes (c.f. Fig 12, [11]). Here, the luminal Cl^−^ entry proceeds exclusively through Cl^−^/HCO_2_^−^ exchanger, which means the coupled transport coefficient for Cl^−^/HCO_3_^−^ is considered to be zero. Also, apical and peritubular membrane permeabilities to H_2_CO_2_ are set at 10% of original reference in Table 1 in S1 File. Luminal concentration for HCO_3_^−^ is considered to stay at 0.004 M, while HCO_3_^−^ peritubular concentration is set to 0.024 M.

Fig 4(a) represents the effect of luminal HCO_3_^−^ concentration on the cellular, tight junction, and total epithelial Cl^−^ fluxes; the total and junctional fluxes illustrate a dramatic decrease in comparison to the cellular flux, which depicts a modest increase. In panel (b), one can see that the general effect of HCO_2_^−^ concentration on overall Cl^−^ absorption for all the fluxes is relatively modest. Panel (c) displays the impact of HCO_2_^−^ concentration, while the luminal and peritubular cell membrane permeability for H_2_CO_2_ was set at 10% of the original value in panel (a) and (b). To maximise the impact of the luminal and peritubular HCO_2_^−^ concentrations for the case of the small H_2_CO_2_ permeability, in [11] the authors assumed that all luminal Cl^−^ entry is through Cl^−^/HCO_2_^−^ exchange. The W-PCT-E demonstrates that small luminal and peritubular cell membrane permeability for H_2_CO_2_ could not sustain any substantial luminal Cl^−^ flux. These results confirm previously published findings according to [11, 25].

We highlight that the ordinate values in Figure 4 are 2-fold greater than Weinstein’s Figure 8 [11]; Weinstein represented a tubule model while here we are describing a cellular epithelial model. To maintain the overall membrane reabsorption, the transporter parameters are multiplied by a factor of two (the transport per unit length needed to be constant).

#### Salt sensitivity

**Fig 5:**
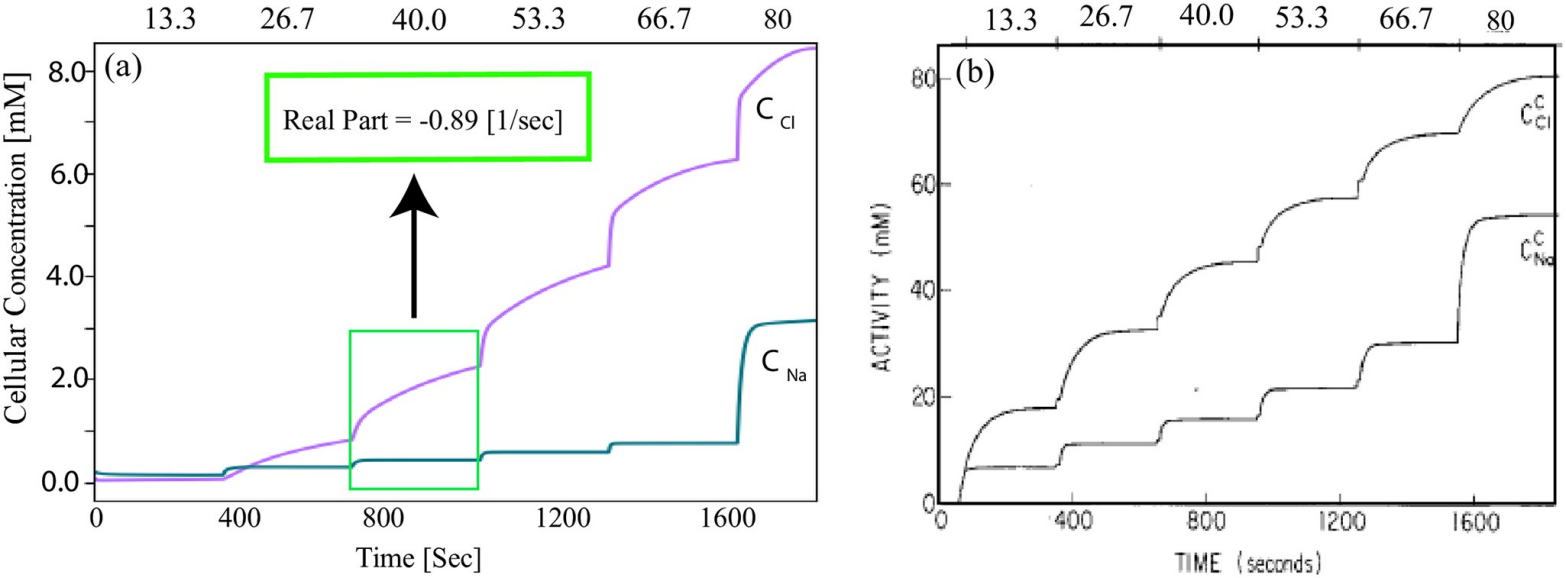
(a) Effect of changes of luminal and peritubular Na^+^ and Cl^−^ concentrations on the cellular system, c.f., Figure 8 in [24] (b).Both peritubular and luminal sodium and chloride concentrations are increased step-wise, identical to the values in the primary paper. The values at the top of the figures show bathing solution activities of Na^+^ and Cl^−^ (in mM).

Next, we test the flexibility, reusability, and reproducibility of the W-PCT-E model by reproducing a simple model of Na^+^ transport in the mammalian urinary bladder to study the salt sensitivity [24]. Here, we aim to design the similar experiment as in Figure 8, [24] where all transporters in the W-PCT-E model are disabled except for the Na^+^/K^+^-ATPase pump. The permeabilities are modified to values much higher than the default values (increased by a factor of six), and solute types are also matched accordingly. The Na^+^ and Cl^−^ concentrations are increased in a step-wise manner, just as with the values in the primary paper. To preserve electroneutrality, there is an increase of 6.65 mM in both peritubular and luminal sodium and chloride concentrations. In the final step of the current experiment, Latta et al. eliminated the effect of the Na^+^/K^+^-ATPase, and here we decreased this effect by a factor of ten. As a consequence, we obtained a step-wise increase in the cellular activities for the primary solutes Na^+^ and Cl^−^, which is displayed in Fig 5(a). Fig 5(b) shows the original result from the primary paper (Figure 8 in [24]). It can be seen that there is a good agreement between the W-PCT-E model and the Na^+^ transport model in the mammalian urinary bladder reported in [24, 26]. We compared these two models qualitatively as there is not much similarity between model parameters and mathematical formulations, although we defined the same configuration for model structures. But we can see a similar behaviour even though the range of the changes in solute concentration is different. We should mention that in the studies cited above, there are only three solutes (Na^+^, K^+^ and Cl^−^), an impermeant monovalent anion to balance electrical charge in the cell and baths, a nonelectrolyte to produce solution osmolarities, and one active transporter, Na^+^/K^+^-ATPase. To see the new set of results, see Fig 5.

### iii. Structural analysis

The goal of the present study was also to create and make available a mathematical model of epithelial transport that is sufficiently flexible to accommodate the investigation of different physiological phenomena in the epithelial system. Here, we performed a structural analysis of the W-PCT-E model to both demonstrate this flexibility and to explore the application of this model to a range of physiological perturbations.

To investigate the effect of each transporter in the W-PCT-E model on the overall behaviour, we performed experiments in which we individually inhibited each of the transporters and compared the total epithelial fluxes. We illustrate some of these results in the following section; the first set of simulations addresses the inhibition of both basal and cell-lateral transporters and the second set of simulations addresses the inhibition of the apical cell transporters.

We should highlight that the following experiments aim to investigate the model behaviour corresponding to either biological or, in some cases, extreme assumptions concerning the model configuration. Considering these assumptions, we can test the models’ limitations, flexibility, or reliability.

For example, we can see a noticeable increase in cell volume in the case of the inhibition of Na^+^/K^+^-ATPase. But, this does not mean the model lacks a self-regulatory system. By inhibiting Na^+^/K^+^-ATPase, we disturb the haemostasis in the epithelial model, causing an accumulation of solutes in the cell, causing a noticeable increase in the cell volume. For more information, see Figs 3 and 4 in S1 File.

**iii.1 Inhibition of peritubular (IS and IE) transporters**. We separately eliminated the Na^+^/K^+^-ATPase and two symporters (K^+^-Cl^−^ and Na^+^-HCO_3_^−^) on both the cell-basal and cell-lateral membranes and observed the resulting membrane fluxes and cellular concentrations. Inhibition of each transporter was accomplished by setting the coupling transport coefficient to zero.

**Fig 6:**
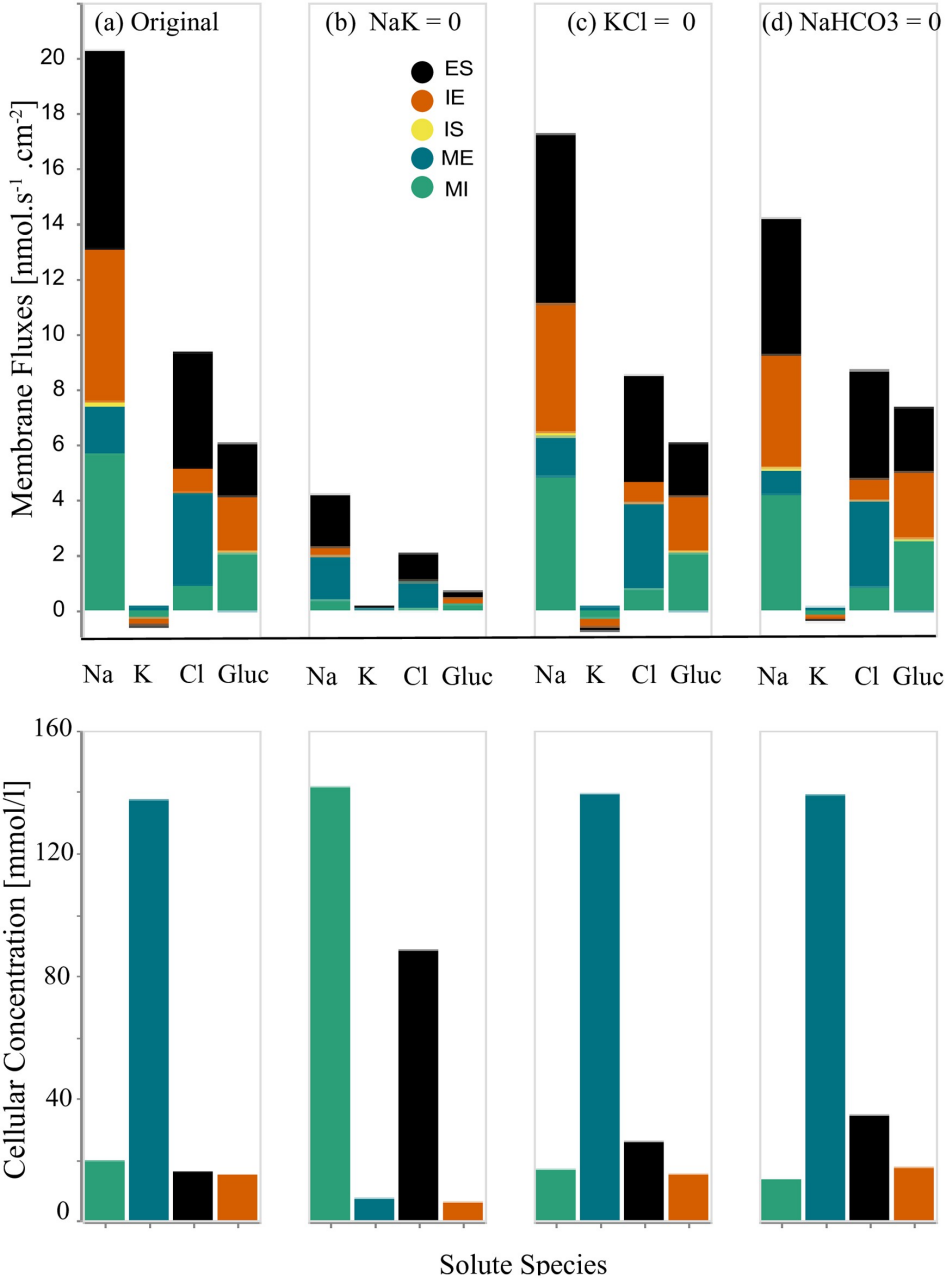
Changes in the membrane fluxes and cellular concentrations due to the inhibition of transporters on the cell-basal and cell-lateral membranes. First row: in each panel, we present four sets of results for four different configurations that depict the total membrane fluxes for the following species: Na^+^, K^+^, Cl^−^, Glucose. The total membrane fluxes include all the membrane activities from five membranes, IS, ME, MI, IE, ES, which are stacked on top of each other. Panel (a) represents the original full model (control configuration). Panel (b) represents scenario due to the Na^+^-K^+^ pump elimination. Panel (c) corresponds to the scenario of K^+^-Cl^−^ elimination, panel (d) is for the inhibition of Na^+^-HCO_3_^−^ transporters. Second row: we illustrate the cellular concentrations corresponding to the related configuration for the same species: Na^+^, K^+^, Cl^−^, Glucose.

We present our results in Fig 6. Panel (a) displays the membrane fluxes (ES, IE, IS, ME, MI) and cellular concentrations for the four primary solutes (Na^+^, K^+^, Cl^−^, Glucose) in the case of the original full W-PCT-E model. Panel (b) represents the results when considering the inhibition of the Na^+^/K^+^-ATPase, from which one can observe a reduction in all the membrane fluxes and notable changes in the cellular solute concentrations; see bottom row in Fig 6(b). There is a considerable reduction in Na^+^ membrane fluxes, which demonstrates the critical role of the Na^+^/K^+^-ATPase in the production of Na^+^ fluxes. Inhibition of the pump stops sodium exit and potassium entry into the cell; thereby, sodium concentration increases (from 19.6 mmol/L to 142.0 mmol/L) while there is a decrease in the cellular potassium concentration (from 137.3 mmol/L to 8.4 mmol/L).

Fig 6(c) illustrates the effect of the inhibition of K^+^-Cl^−^ transporter on the W-PCT-E model activity. One can observe a decrease in total fluxes for both sodium and chloride. Although there is a decrease in the sodium concentration, there is an increase in potassium and chloride concentrations. Fig 6(d) illustrates the response of the W-PCT-E model in the case of elimination of Na^+^-HCO_3_^−^ transporter. There is a significant decrease in sodium total flux, accompanied by notable growth in glucose fluxes. While there is a decrease in the sodium concentration, one can see an increase in other solute concentrations. To better understand the underlying mechanisms of the W-PCT-E model responses to these structural changes, we narrow our focus to sodium. We then study how the elimination of Na^+^/K^+^-ATPase can affect the sodium fluxes across the epithelial membrane. We need to clarify that there are no transporters across the tight junction and interspace basement and the corresponding fluxes across these membranes are either convective or passive. While in the cell-basal, cell-lateral, and apical cell membrane, the passive and convective fluxes are negligible, for the reflection and permeability coefficient values are considered to be very small, see Table 1. Electrochemical fluxes represent the primary source of fluxes across the cell-basal, cell-lateral, and apical cell membranes.

**Fig 7:**
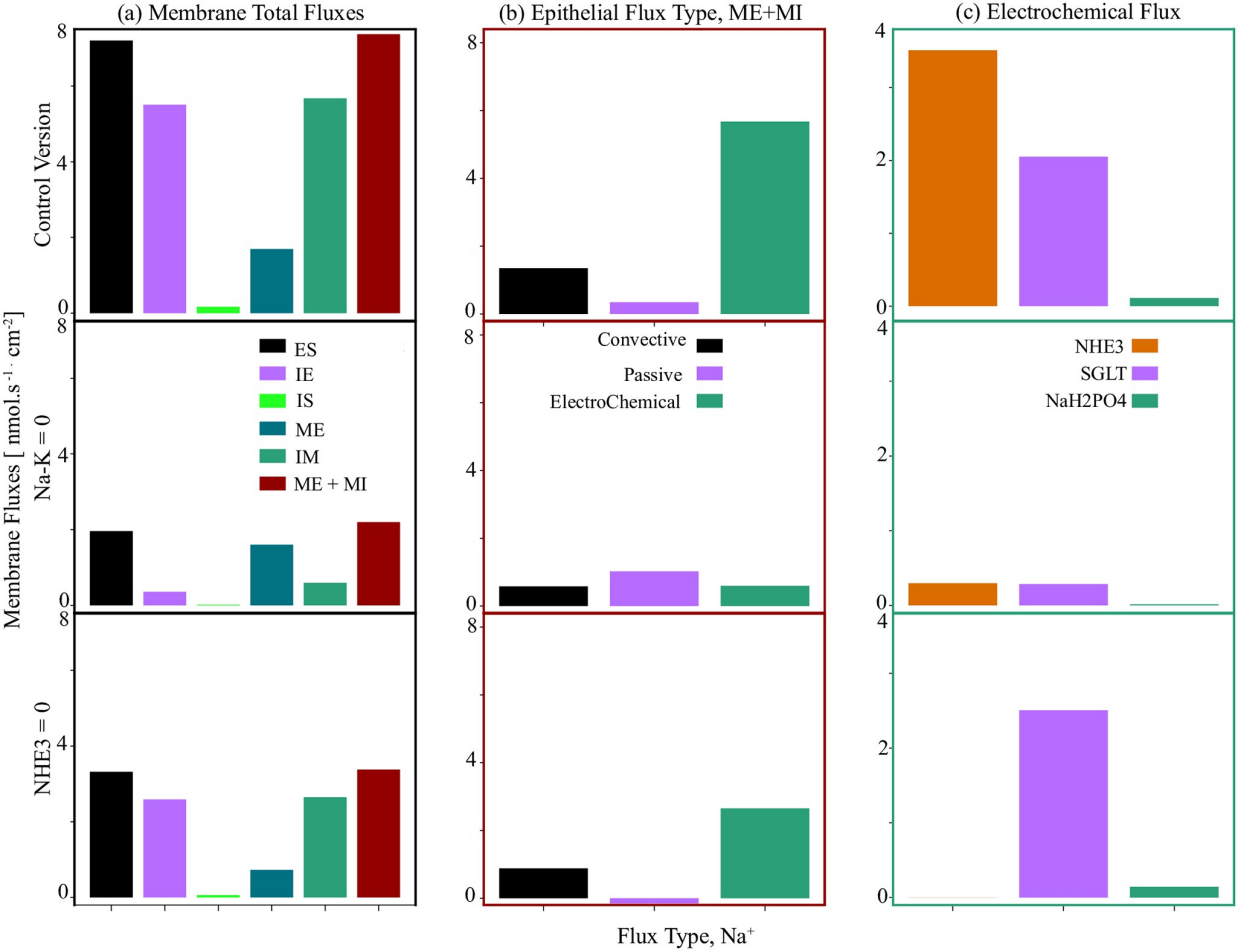
Total epithelial sodium fluxes and the contribution of various sodium flux types. Panel (a) illustrates the different membrane fluxes. Panel (b) presents the epithelial sodium fluxes classified into convective, passive and electrochemical types. Panel (c) details the electrodiffusive activities into their segments: NHE3, SGLT, and Na^+^-H_2_PO_4_^−^. The first row corresponds to the full (control) model, the second row is for the case of elimination of the Na^+^/K^+^-ATPase, and in the third row the results are for the elimination of NHE3.

Fig 7(a) features all different sodium membrane fluxes. In Fig 7(b), we narrow our focus down to sodium fluxes on the epithelial membrane (summation of the tight junction and apical cell membrane activities) and its components. The epithelial activities subdivide into three components: convective, passive, and electrochemical activities. In Fig 7c, we subdivide the electrodiffusive coupled fluxes into their segments, which are NHE3, SGLT, and Na^+^-H_2_PO_4_^−^ transporters.

The first row in Fig 7 represents the sodium fluxes for the full W-PCT-E model, considered as the control version. In the second row, we illustrate the sodium fluxes due to the elimination of the Na^+^/K^+^-ATPase. By a simple comparison between the first and second rows, one can see that the membrane activities drop considerably after the Na^+^/K^+^-ATPase inhibition; as an example, the total epithelial sodium flux decreases from 7.39 nmol.s^-1^.cm^-2^ to 2.19 nmol.s^-1^.cm^-2^, as seen by comparing the first and second rows in Fig 7(a). To gain insight into the exacerbated decline in epithelial activity, we further divide the epithelial activity into its components, as seen in the second row of Fig 7(b). One can observe a marked drop in the convective fluxes (from 1.348 nmol.s^-1^.cm^-2^ to 0.58 nmol.s^-1^.cm^-2^), which is due to the notable drop in the tight junction water fluxes (from 16.7 nmol.s^-1^.cm^-2^ to 7.21 nmol.s^-1^.cm^-2^), see Eq (37) and Fig 7(b) (first and second rows). In contrast, there is an intensification in passive activities (from 0.34 nmol.s^-1^.cm^-2^ to 1.02 nmol.s^-1^.cm^-2^, which is due to the changes of the normalised electrical potential differences, which appear in the form of linear and exponential expressions, as described by Eq (39), and seen in Fig 7(c) (first and second rows).

The total electrochemical flux falls from 5.67 nmol.s^-1^.cm^-2^ to 0.59 nmol.s^-1^.cm^-2^, see Fig 7(b). We study the electrochemical fluxes in the last panel by visualising the relevant components and their changes individually: NHE3, SGLT, and Na^+^-H_2_PO_4_^−^. Due to the inhibition of the pumps, there is a significant increase in the sodium cellular concentration (from 19.6 mmol/L to 142.7 mmol/L), which notably decreases the sodium concentration gradient between the lumen (140 mmol/L) and cell, decreasing the electrochemical potential difference of sodium across the apical cell membrane. This, in turn, decreases the flux of the transporters related to the production of sodium fluxes, namely: NHE3 (from 2.05 nmol.s^-1^.cm^-2^ to 0.29 nmol.s^-1^.cm^-2^), SGLT (from 2.05 nmol.s^-1^.cm^-2^ to 0.28 nmol.s^-1^.cm^-2^), and Na^+^-H_2_PO_4_^−^ (from 0.11 nmol.s^-1^.cm^-2^ to 0.0143 nmol.s^-1^.cm^-2^).

Here, we aim to highlight the flexibility of the W-PCT-E model, as the user can have a better insight into the system behaviour due to the elimination or cooperation of transporters by simply turning them on or off.

Because of the flexibility of the W-PCT-E model, there is future opportunity for similar analyses to describe the system behaviour due to the elimination of other transporters and their impact on the different solutes.

Here, we applied extreme assumptions to study and understand the performance of the model under these conditions (such as changes in model configurations and model parameters). However, one should not expect to see biological behaviour due to these extreme assumptions. As mentioned in Subsection iii, there is an increase in cell volume due to the elimination of Na^+^/K^+^-ATPase; for more detailed information, see Figs 3 and 4 in S1 File.

**Fig 8:**
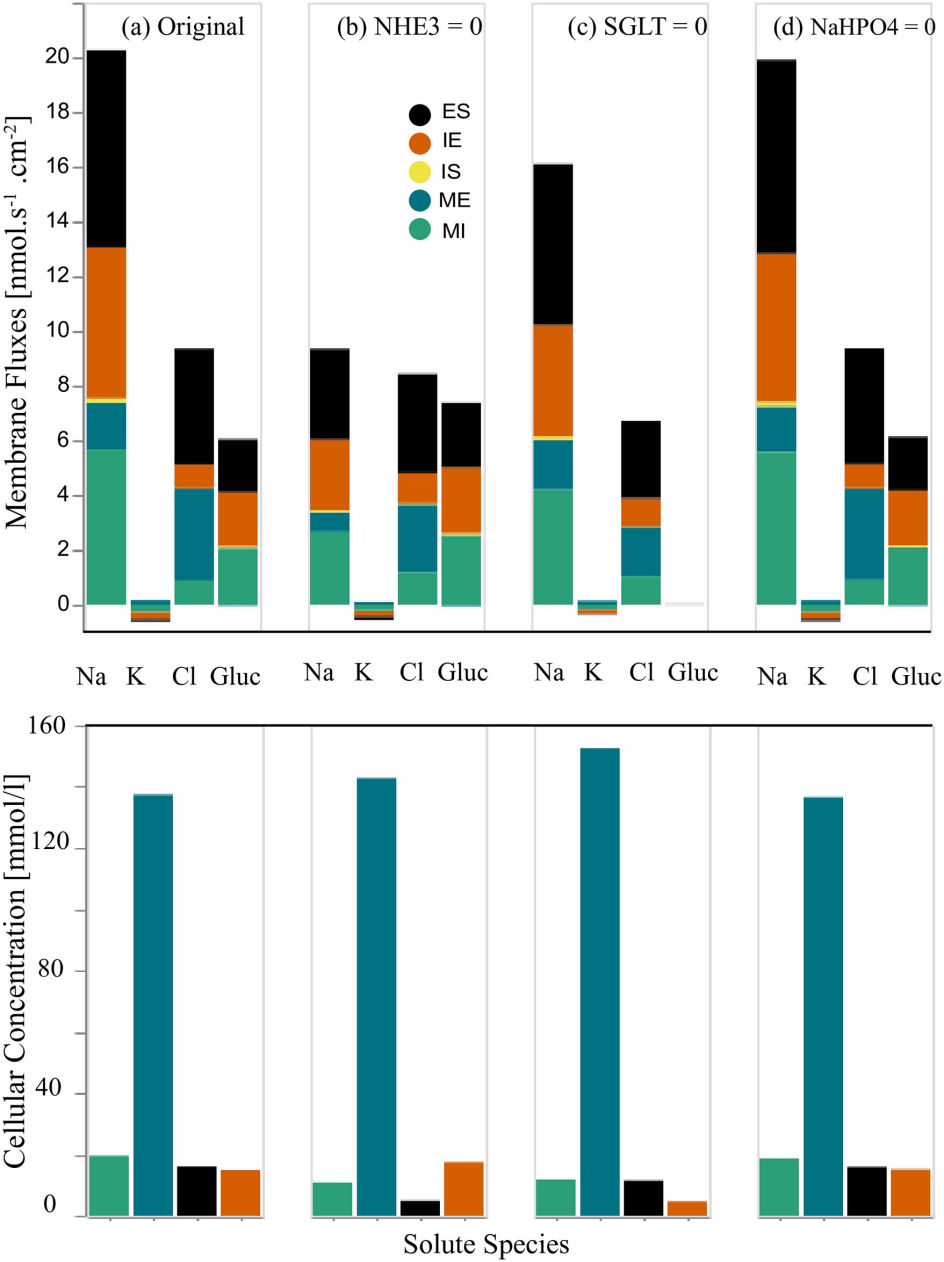
Changes in the membrane fluxes and cellular concenting rations due to the inhibition of transporters on the apical cell membrane. In the first row, in each panel, we present four sets of results for four different configurations that depict the total membrane fluxes for the following species: Na^+^, K, Cl, Glucose. The total membrane fluxes include all the membrane activities from five membranes, IS, ME, MI, IE, ES, which are stacked on top of each other. Panel (a) represents the original full model (control scenario). Panel (b) represents flux changes due to the NHE3 elimination. Panel (c) is for the removal of the SGLT, and panel (d) due to inhibition of Na^+^-H_2_PO_4_^−^ transporters. In the second row, we illustrate the cellular concentrations for the same species: Na^+^, K^+^, Cl^−^, Glucose in each scenario.

**iii.2 Inhibition of apical membrane (MI) transporters**. In this section, we separately eliminate the NHE3 antiporter and apical symporters (SGLT and Na^+^-H_2_PO_4_^−^) and then we study the behaviour of the W-PCT-E model by analysing the results for membrane fluxes and cellular concentrations relative to each scenario. In Fig 8, we present the membrane fluxes in the first row and the cellular concentrations in the second row. Fig 8(a) displays the membrane fluxes (ES, IE, IS, ME, MI) and cellular concentrations for the four primary solutes (Na^+^, K^+^, Cl^−^, Glucose) in the case of the original full W-PCT-E model. Fig 8(b) represents the membrane fluxes and cellular concentrations due to the inhibition of the NHE3. In panels (c) and (d), we illustrate the effect of the inhibition of SGLT and Na^+^-H_2_PO_4_^−^ transporters on the W-PCT-E model responses, respectively.

By inhibiting NHE3, one can observe a notable decrease not only in sodium membrane fluxes, but also in cellular sodium and chloride concentrations. While there is an increase in glucose membrane fluxes and cellular glucose concentration, as seen in Fig 8(b).

By eliminating SGLT, we consider the total absence of glucose membrane fluxes which is consistent with the model structure as there are no other sources to produce either convective or passive fluxes of glucose. There is a decrease in both Na^+^ and Cl^−^ fluxes; while cellular sodium and chloride concentrations depict decreases, potassium and glucose concentrations demonstrate increases, as can be seen in Fig 8(c).

Removal of Na^+^-H_2_PO_4_ does not show any significant changes neither in epithelial fluxes nor cellular concentrations in any of the four primary solutes, as clearly shown in Fig 8(d).

Here, we focus on the sodium fluxes, as NHE3 is the primary source of sodium fluxes in the epithelial model. NHE3 inhibition stops the exit of hydrogen and ammonia from the cell and the entry of sodium into the cell. Sodium concentration drops from 19.6 mmol/L to 10.5 mmol/L, which demonstrates the role of NHE3 in the production of Na^+^ fluxes.

To have a better understanding of Fig 8(b) and a deeper insight into the underlying mechanisms of NHE3 and its effect on the sodium fluxes, we study all sources of sodium fluxes, and the results are featured in the third row in Fig 7.

After inhibition of NHE3 in Fig 7 (third row), sodium activities in epithelial membranes (ME and MI) drop considerably (the total activity drops from 7.39 nmol.s^-1^.cm^-2^ to 3.37 nmol.s^-1^.cm^-2^, Fig 7(a)). To better understand the origin of these changes, we further divide the epithelial sodium fluxes into convective, passive, and electrochemical component fluxes, as seen in Fig 7(b) (third row).

As we mentioned before, convective and passive fluxes in the epithelial membrane are mainly through the tight junction. In Fig 7(b) (third row), one can observe a notable drop in the convective fluxes (from 1.348 nmol.s^-1^.cm^-2^ to 0.88 nmol.s^-1^.cm^-2^), which is mostly due to the reduction in the water fluxes (from 16.7 nmol.s^-1^.cm^-2^ to 11.01 nmol.s^-1^.cm^-2^), see Eq (37).

There is a slight reduction in passive fluxes accompanied by changes in direction (from 0.34 nmol.s^-1^.cm^-2^ to −0.15 nmol.s^-1^.cm^-2^). To explain the decrease in the passive fluxes, we need to bring to attention that the main driving force for the passive fluxes is not only the normalised electrical potential differences (which are in the form of linear and exponential components, see Eq (39)) but also solute concentrations. The total electrochemical flux across the epithelial membrane falls from 5.67 nmol.s^-1^.cm^-2^ to 2.64 nmol.s^-1^.cm^-2^, see Fig 7(b). We then investigate the coupled transport fluxes in Fig 7(c) by visualising the components individually: NHE3, SGLT, and Na^+^-H_2_PO_4_^−^. Sodium fluxes for NHE3 dropped to zero as a result of inhibition. Due to changes in sodium cellular concentration (from 19.6 mmol/L to 10.7 mmol/L), the sodium concentration gradient between the lumen (140 mmol/L) and cell increases notably, increasing the electrochemical potential difference of sodium across the apical cell membrane, which increases the activity of transporters related to the production of sodium fluxes (SGLT from 2.05 nmol.s^-1^.cm^-2^ to 2.50 nmol.s^-1^.cm^-2^, and Na^+^-H_2_PO_4_^−^ from 0.11 nmol.s^-1^.cm^-2^ to 0.143 nmol.s^-1^.cm^-2^).

## VII. Discussion

Mathematical modelling provides a tool for investigating complex physiological phenomena that is hardly accessible to experimentation. A comprehensive example of these tools is the work done by Alan Weinstein in the field of renal modelling. Weinstein has provided a valuable resource for mechanistic modelling of many aspects of renal function through many publications [9–16, for example]. However, existing mathematical models of epithelial transporters are, in general, not readily findable, accessible, interoperable, or reusable (FAIR) [27] for researchers who are new to this field. Here, we want to provide a well-tested reusable implementation of Weinstein epithelial PCT models, collated into a single consistent implementation that we believe encourages reuse.

We have presented here what we believe to be a comprehensive and FAIR epithelial model for the PCT of the renal nephron. This model encapsulates and recapitulates the seminal work performed by Weinstein and colleagues [9–13] over many years. In the case of model reproducibility, we have demonstrated that the W-PCT-E model reported here can reproduce many different aspects from related or earlier works. We chose three exemplars [10, 11, 24], the constant parameters and boundary conditions tuned according to the model of interest. In all cases, we observed a close agreement between the W-PCT-E simulation outcomes and the results reported in previous works, see Section ii (Table 1, Figs 4 and 5).

To show the flexibility of the implementation of the W-PCT-E through the application of structural analysis, we investigated the impact of each transporter on the W-PCT-E model responses (total fluxes and cellular concentrations) through the inhibition of each transporter, see Section iii (Figs 6–8).

To further study the limitations and performance of our model, we examined it under some extreme conditions. Due to the non-physiological nature of these conditions, one should not expect to observe real-world physiological behaviours. Here, we aim to demonstrate the mathematical feasibility of the W-PCT-E model configuration by removing or appending different elements. As an example, there is an increase in the cell volume due to the elimination of Na^+^/K^+^-ATPase (Fig 6).

To demonstrate the comprehensiveness and flexibility of the W-PCT-E model, we now briefly explore various physiological phenomena using our model.

### Clinical studies have shown that excess glucose in the cell and bloodstream is associated with Type II Diabetes (T2D) [28–36]

The inhibition of SGLT for the treatment of T2D [37, 38] has shown improvement of glycemic control and T2D [39–41]. According to the W-PCT-E model, the inhibition of the SGLT transporters decreases the cellular Na^+^ and glucose fluxes which decrease the cellular concentration of glucose, see Fig 8(c). The model also predicts a decrease in interspace concentration of glucose, which would lead to less glucose available for reabsorbtion into the blood. Thus, demonstrating a clear consistency between the W-PCT-E and the clinical findings regarding the inhibition of SGLT and improvement of T2D.

### Experimental reports illustrate that NHE3 residing in the apical membrane mediates transcellular reabsorption of Na^+^ and fluid reabsorption [42, 43], and show that NHE3 deficiency can cause a reduction in Na^+^ reabsorption [8, 44]

These observations put renal Na^+^ reabsorption via NHE3 in a central position in the development and control of salt loading- and volume expansion-mediated hypertension [45]. We performed some simulation experiments to check that our W-PCT-E model is consistent with these findings by inhibiting NHE3. In doing so, we observed that inhibition of the apical NHE3 decreases the intracellular concentration of HCO_3_^−^ and HCO_2_^−^, which in turn is transported via the apical Cl^−^/HCO_3_^−^ or Cl^−^/HCO_2_^−^ exchangers. The inhibited apical NHE3 and the Cl^−^/base exchangers work in parallel and produce net Na^+^ reduction and Cl^−^ reabsorption in the proximal tubule, see Fig 1(b)–1(e) in S1 File (cellular concentrations) and Fig 1(F) in S1 File (cell volume). These findings are also consistent with other studies demonstrating that the inhibition of either the apical NHE3 and Cl^−^/base exchangers inhibits net Na^+^ and Cl^−^ absorption in the proximal tubule [11, 46–49].

### Experimental results reveal that a global knockout of NHE3 gene on the proximal tubule reduces water fluxes and HCO_3_^−^ absorption [50]

The W-PCT-E model demonstrates that the apical NHE3 exchanger can inhibit HCO_3_^−^ flexes in the proximal epithelial model. We designed a set of experiments, in which we decreased the coupled transport coefficient for NHE3, see Fig 1 in S1 File. We observed that the inhibition of NHE3 lowers the Cl^−^/HCO_3_^−^ and reduces the cell volume. These findings are consistent with the earlier findings [11, 50–52].

### Experimental reports show that there is a coordination between basolateral Na^+^/K^+^-ATPase and apical NHE3 activities; they simultaneously regulate the Na^+^ transport which can be the cause of inhibition or activation of transepithelial Na^+^ transport [31, 45, 53–55]

In the W-PCT-E model, one can see that there is strong coordination between basolateral Na^+^/K^+^-ATPase and apical NHE3 activities; inhibition of NHE3 (Na^+^/K^+^-ATPase) can cause the inhibition of Na^+^/K^+^-ATPase (NHE3). We can conclude from this observation that in the W-PCT-E model, sodium is primarily reabsorbed via NHE3 which is regulated by Na^+^/K^+^-ATPase, see Fig 2 in S1 File.

### The kidney plays a critical role in the regulation of body electrolyte and fluid balance, primarily occurring in the proximal tubule segment of the nephron [56–58]

A balance of body extracellular electrolyte composition and fluid volume is essential for all animals and humans to survive. Either excess or shortage of crucial extracellular electrolytes or overall fluid volume may lead to disturbance of blood pressure and abnormalities in cellular functions, including cell volume [58–62]. The W-PCT-E model can capture the impact of modulation of different transporters on the fluid balance and cellular volume. For example, with the inhibition of basal-cell Na^+^/K^+^-ATPase or the activation of apical NHE3 or SGLT, we observed increase in the cell volume. Such observations are consistent with previous modelling approaches and experimental reports [31, 60, 62, 63]. In subsection iii, we mentioned that there is an increase in cell volume due to the elimination of Na^+^/K^+^-ATPase; we also added Figs 3 and 4 in S1 File.

The implementation of the present epithelial transport model to support the composition and parameterisation of the proximal tubule epithelial system (the W-PCT-E model) is available on Github under a license which allows open and unrestricted reuse. The implementation is in Python (version 3) to make it broadly accessible. As demonstrated above, the implementation is sufficiently flexible and configurable to support the generation of different epithelial models. Users need only to extract the required constituent transporter modules from the available repository and then integrate them into the desired configurations. The boundary conditions need to be changed according to the epithelium of interest. In sharing the model implementation between the authors of this manuscript, we tested for reusability as we each work with W-PCT-E model.

## VII. Conclusions

We believe that the time is now right to develop a reproducible and FAIR virtual nephron. Here, we term this the iNephron. It is critical to ensure that the capabilities of published models are captured while being sufficiently flexible and configurable to support the generation of novel models to investigate specific scientific or clinical questions. To achieve this, iNephron will provide a tool to investigate the fundamental mechanisms involved in hypertension, diabetes, and many other kidney diseases. Furthermore, iNephron will be developed and shared following established best practices in open and reproducible science to guarantee that the scientific community can benefit and extend from this work to improve our collective understanding of these diseases.

The work we presented here is our first step toward achieving the iNephron. As we look to grow the repository of available transporter modules, we also look to better follow FAIR principles [27, 64], and move toward a standards-based repository of reusable modules (e.g., [65]). To ensure the composed models are meaningful and that model composition can occur reliably, we are planning to migrate our repository of reusable modules to ensure thermodynamic consistency [3–7]. This is a requirement for the arbitrary composition of models needed for iNephron.

As we have shown, the W-PCT-E model is actually a generic epithelial model which is flexible and configurable to support the generation of different epithelial models, where the user is able to meet their design requirements, easing the process for “getting started” with a novel modelling study. All one needs is to provide the model with a set of transporters and boundary conditions appropriate for the epithelial model of interest. Additionally, by establishing a comprehensive ability to perform sensitivity analyses, we provided tools by which future users are able to test their own additions or modifications of this model with confidence. Our testing, summarised in this manuscript with more detailed findings in the supplemental material, shows that our model behaves as expected in physiological terms.

## Supporting information

S1 File (ZIP)

## References

[1] Cuellar AA, Lloyd CM, Nielsen PF, Bullivant DP, Nickerson DP, Hunter PJ. An Overview of CellML 1.1, a Biological Model Description Language. SIMULATION. 2003;79(12):740–747. doi: 10.1177/0037549703040939

[2] Clerx M, Cooling MT, Cooper J, Garny A, Moyle K, Nickerson DP, et al. CellML 2.0. Journal of Integrative Bioinformatics. 2020;17(2-3). doi: 10.1515/jib-2020-0021 32759406 PMC7756617

[3] Pan M, Gawthrop PJ, Tran K, Cursons J, Crampin tt. A thermodynamic framework for modelling membrane transporters. Journal of Theoretical Biology. 2019;481:10–23. doi: 10.1016/j.jtbi.2018.09.034 30273576

[4] Gawthrop PJ, Crampin tt. Energy-based analysis of biomolecular pathways. Proceedings of the Royal Society A: Mathematical, Physical and Engineering Sciences. 2017;473(2202):20160825. doi: 10.1098/rspa.2016.0825 28690404 PMC5493942

[5] Gawthrop PJ, Crampin tt. Modular bond-graph modelling and analysis of biomolecular systems. IET Systems Biology. 2016;10(5):187–201. doi: 10.1049/iet-syb.2015.0083 27762233 PMC8687434

[6] Gawthrop PJ, Crampin tt. Energy-based analysis of biochemical cycles using bond graphs. Proceedings of the Royal Society A: Mathematical, Physical and Engineering Sciences. 2014;470(2171):20140459. doi: 10.1098/rspa.2014.0459 25383030 PMC4197480

[7] Gawthrop PJ, Cursons J, Crampin tt. Hierarchical bond graph modelling of biochemical networks. Proceedings of the Royal Society A: Mathematical, Physical and Engineering Sciences. 2015;471(2184):20150642. doi: 10.1098/rspa.2015.0642

[8] Lorenz JN, Schultheis PJ, Traynor T, Shull GE, Schnermann J. Micropuncture analysis of single-nephron function in NHE3-deficient mice. American Journal of Physiology-Renal Physiology. 1999;277(3):F447–F453. doi: 10.1152/ajprenal.1999.277.3.F447 10484528

[9] Weinstein AM. Modeling the proximal tubule: complications of the paracellular pathway. American Journal of Physiology-Renal Physiology. 1988;254(3):F297–F305. doi: 10.1152/ajprenal.1988.254.3.F297 3279817

[10] Weinstein AM. A mathematical model of the rat proximal tubule. American Journal of Physiology-Renal Physiology. 1986;250(5):F860–F873. doi: 10.1152/ajprenal.1986.250.5.F860 3706537

[11] Weinstein AM. Chloride transport in a mathematical model of the rat proximal tubule. American Journal of Physiology-Renal Physiology. 1992;263(5):F784–F798. doi: 10.1152/ajprenal.1992.263.5.F784 1443169

[12] Weinstein AM, Weinbaum S, Duan Y, Du Z, Yan Q, Wang T. Flow-dependent transport in a mathematical model of rat proximal tubule. American Journal of Physiology-Renal Physiology. 2007;292(4):F1164–F1181. doi: 10.1152/ajprenal.00392.2006 17213461

[13] Weinstein AM. Potassium deprivation: a systems approach. American Journal of Physiology-Renal Physiology. 2011;301(5):F967–F968. doi: 10.1152/ajprenal.00430.2011 21849489 PMC3213898

[14] Weinstein AM. A mathematical model of rat proximal tubule and loop of Henle. American Journal of Physiology-Renal Physiology. 2015;308(10):F1076–F1097. doi: 10.1152/ajprenal.00504.2014 25694479 PMC4437000

[15] Weinstein AM. A mathematical model of the rat nephron: glucose transport. American Journal of Physiology-Renal Physiology. 2015;308(10):F1098–F1118. doi: 10.1152/ajprenal.00505.2014 25694480 PMC4437004

[16] Weinstein AM. A mathematical model of the rat kidney: K+-induced natriuresis. American Journal of Physiology-Renal Physiology. 2017;312(6):F925–F950. doi: 10.1152/ajprenal.00536.2016 28179254 PMC6148314

[17] Edwards A, Bonny O. A model of calcium transport and regulation in the proximal tubule. American Journal of Physiology-Renal Physiology. 2018;315(4):F942–F953. doi: 10.1152/ajprenal.00129.2018 29846115 PMC6230728

[18] Layton AT, Vallon V, Edwards A. Modeling oxygen consumption in the proximal tubule: effects of NHE and SGLT2 inhibition. American Journal of Physiology-Renal Physiology. 2015;308(12):F1343–F1357. doi: 10.1152/ajprenal.00007.2015 25855513 PMC4469883

[19] Layton AT. Recent advances in renal hypoxia: Insights from bench experiments and computer simulations. American Journal of Physiology-Renal Physiology. 2016;311(1):F162–F165. doi: 10.1152/ajprenal.00228.2016 27147670 PMC4967163

[20] Weinstein AM. A kinetically defined Na^+^/H^+^ antiporter within a mathematical model of the rat proximal tubule. The Journal of General Physiology. 1995;105(5):617–641. doi: 10.1085/jgp.105.5.617 7658195 PMC2216949

[21] Weinstein AM, Sontag ED. Modeling proximal tubule cell homeostasis: tracking changes in luminal flow. Bulletin of Mathematical Biology. 2009;71(6):1285–1322. doi: 10.1007/s11538-009-9402-1 19280266 PMC2793416

[22] Hodgkin AL, Katz B. The effect of sodium ions on the electrical activity of the giant axon of the squid. The Journal of Physiology. 1949;108(1):37–77. doi: 10.1113/jphysiol.1949.sp004310 18128147 PMC1392331

[23] Noroozbabaee L, Steyn-Ross D, Steyn-Ross ML, Sleigh JW. Analysis of the Hindriks and van Putten model for propofol anesthesia: Limitations and extensions. NeuroImage. 2021;227:117633. doi: 10.1016/j.neuroimage.2020.117633 33316393

[24] Latta R, Clausen C, Moore LC. General method for the derivation and numerical solution of epithelial transport models. J Membrane Biol. 1984;82(1):67–82. doi: 10.1007/BF01870733 6502699

[25] Preisig PA, Ives HE, Cragoe EJ, Alpern RJ, Rector FC. Role of the Na+/H+ antiporter in rat proximal tubule bicarbonate absorption. J Clin Invest. 1987;80(4):970–978. doi: 10.1172/JCI113190 2888788 PMC442334

[26] Lewis SA, Eaton DC, Clausen C, Diamond JM. Nystatin as a probe for investigating the electrical properties of a tight epithelium. The Journal of General Physiology. 1977;70(4):427–440. doi: 10.1085/jgp.70.4.427 915470 PMC2228507

[27] Wilkinson MD, Dumontier M, Aalbersberg IJ, Appleton G, Axton M, Baak A, et al. The FAIR Guiding Principles for scientific data management and stewardship. Scientific Data. 2016;3(1):1–9. doi: 10.1038/sdata.2016.18 26978244 PMC4792175

[28] Vallon V, Platt KA, Cunard R, Schroth J, Whaley J, Thomson SC, et al. SGLT2 mediates glucose reabsorption in the early proximal tubule. Journal of the American Society of Nephrology. 2011;22(1):104–112. doi: 10.1681/ASN.2010030246 20616166 PMC3014039

[29] Vallon V, Gerasimova M, Rose MA, Masuda T, Satriano J, Mayoux E, et al. SGLT2 inhibitor empagliflozin reduces renal growth and albuminuria in proportion to hyperglycemia and prevents glomerular hyperfiltration in diabetic Akita mice. American Journal of Physiology-Renal Physiology. 2014;306(2):F194–F204. doi: 10.1152/ajprenal.00520.2013 24226524 PMC3920018

[30] Pessoa TD, Campos LCG, Carraro-Lacroix L, Girardi AC, Malnic G. Functional role of glucose metabolism, osmotic stress, and sodium-glucose cotransporter isoform-mediated transport on Na+/H+ exchanger isoform 3 activity in the renal proximal tubule. Journal of the American Society of Nephrology. 2014;25(9):2028–2039. doi: 10.1681/ASN.2013060588 24652792 PMC4147971

[31] Zhuo JL, Li XC. Proximal nephron. Comprehensive Physiology. 2013;3(3):1079–1123. doi: 10.1002/cphy.c110061 23897681 PMC3760239

[32] Ghezzi C, Wright EM. Regulation of the human Na+-dependent glucose cotransporter hSGLT2. American Journal of Physiology-Cell Physiology. 2012;303(3):C348–C354. doi: 10.1152/ajpcell.00115.2012 22673616 PMC3423026

[33] Wright EM, Loo DD, Hirayama BA. Biology of human sodium glucose transporters. Physiological reviews. 2011;91(2):733–794. doi: 10.1152/physrev.00055.2009 21527736

[34] Oku A, Ueta K, Arakawa K, Ishihara T, Nawano M, Kuronuma Y, et al. T-1095, an inhibitor of renal Na^+^-glucose cotransporters, may provide a novel approach to treating diabetes. Diabetes. 1999;48(9):1794–1800. doi: 10.2337/diabetes.48.9.1794 10480610

[35] Linden KC, DeHaan CL, Zhang Y, Glowacka S, Cox AJ, Kelly DJ, et al. Renal expression and localization of the facilitative glucose transporters GLUT1 and GLUT12 in animal models of hypertension and diabetic nephropathy. American Journal of Physiology-Renal Physiology. 2006;290(1):F205–F213. doi: 10.1152/ajprenal.00237.2004 16091581

[36] Marks J, Carvou NJ, Debnam ES, Srai SK, Unwin RJ. Diabetes increases facilitative glucose uptake and GLUT2 expression at the rat proximal tubule brush border membrane. The Journal of Physiology. 2003;553(1):137–145. doi: 10.1113/jphysiol.2003.046268 12963802 PMC2343472

[37] Scheen AJ. Pharmacodynamics, efficacy and safety of sodium–glucose co-transporter type 2 (SGLT2) inhibitors for the treatment of type 2 diabetes mellitus. Drugs. 2015;75(1):33–59. doi: 10.1007/s40265-014-0337-y 25488697

[38] Vallon V. The mechanisms and therapeutic potential of SGLT2 inhibitors in diabetes mellitus. Annual review of medicine. 2015;66:255–270. doi: 10.1146/annurev-med-051013-110046 25341005

[39] Fioretto P, Zambon A, Rossato M, Busetto L, Vettor R. SGLT2 inhibitors and the diabetic kidney. Diabetes Care. 2016;39(Supplement 2):S165–S171. doi: 10.2337/dcS15-3006 27440829

[40] Škrtic M, Cherney DZ. Sodium–glucose cotransporter-2 inhibition and the potential for renal protection in diabetic nephropathy. Current opinion in nephrology and hypertension. 2015;24(1):96–103. doi: 10.1097/MNH.0000000000000084 25470017

[41] Kalra S, Singh V, Nagrale D. Sodium-glucose cotransporter-2 inhibition and the glomerulus: a review. Advances in therapy. 2016;33(9):1502–1518. doi: 10.1007/s12325-016-0379-5 27423646 PMC5020120

[42] Biemesderfer D, Pizzonia J, Abu-Alfa A, Exner M, Reilly R, Igarashi P, et al. NHE3: a Na+/H+ exchanger isoform of renal brush border. American Journal of Physiology-Renal Physiology. 1993;265(5):F736–F742. doi: 10.1152/ajprenal.1993.265.5.F736 8238556

[43] Amemiya M, Loffing J, Lötscher M, Kaissling B, Alpern RJ, Moe OW. Expression of NHE-3 in the apical membrane of rat renal proximal tubule and thick ascending limb. Kidney international. 1995;48(4):1206–1215. doi: 10.1038/ki.1995.404 8569082

[44] Ledoussal C, Lorenz JN, Nieman ML, Soleimani M, Schultheis PJ, Shull GE. Renal salt wasting in mice lacking NHE3 Na+/H+ exchanger but not in mice lacking NHE2. American Journal of Physiology-Renal Physiology. 2001;281(4):F718–F727. doi: 10.1152/ajprenal.2001.281.4.F718 11553519

[45] Liu J, Yan Y, Shapiro JI. The Na/K-ATPase Signaling Regulates Natriuresis in Renal Proximal Tubule. Innovative Bioanalysis. 2020;. 33118839

[46] Preisig P, Rector F Jr. Role of Na+-H+ antiport in rat proximal tubule NaCl absorption. American Journal of Physiology-Renal Physiology. 1988;255(3):F461–F465. doi: 10.1152/ajprenal.1988.255.3.F461 2843052

[47] Lucci MS, Warnock DG, et al. Effects of anion-transport inhibitors on NaCl reabsorption in the rat superficial proximal convoluted tubule. The Journal of clinical investigation. 1979;64(2):570–579. doi: 10.1172/JCI109495 457869 PMC372152

[48] Baum M, Berry C, et al. Evidence for neutral transcellular NaCl transport and neutral basolateral chloride exit in the rabbit proximal convoluted tubule. The Journal of clinical investigation. 1984;74(1):205–211. doi: 10.1172/JCI111403 6736248 PMC425202

[49] Thomas SR, Mikulecky DC. A network thermodynamic model of salt and water flow across the kidney proximal tubule. American Journal of Physiology-Renal Physiology. 1978;235(6):F638–F648. doi: 10.1152/ajprenal.1978.235.6.F638 736148

[50] Wang Z, Petrovic S, Mann E, Soleimani M. Identification of an apical Cl-/HCO3-exchanger in the small intestine. American Journal of Physiology-Gastrointestinal and Liver Physiology. 2002;282(3):G573–G579. doi: 10.1152/ajpgi.00338.2001 11842009

[51] Petrovic S, Barone S, Weinstein AM, Soleimani M. Activation of The Apical Na/H Exchanger NHE3 By Formate: A Basis of Enhanced Fluid and Electrolyte Reabsorption By Formate in the Kidney Proximal Tubule. Pres Am J Physiol Renal Physiol. 2004;. doi: 10.1152/ajprenal.00400.2003 15082449

[52] Onishi A, Fu Y, Darshi M, Crespo-Masip M, Huang W, Song P, et al. Effect of renal tubule-specific knockdown of the Na+/H+ exchanger NHE3 in Akita diabetic mice. American Journal of Physiology-Renal Physiology. 2019;317(2):F419–F434. doi: 10.1152/ajprenal.00497.2018 31166707 PMC6732454

[53] Li XC, Shull GE, Miguel-Qin E, Zhuo JL. Role of the Na+/H+ exchanger 3 in angiotensin II-induced hypertension. Physiological genomics. 2015;47(10):479–487. doi: 10.1152/physiolgenomics.00056.2015 26242933 PMC4593829

[54] McDonough AA. Mechanisms of proximal tubule sodium transport regulation that link extracellular fluid volume and blood pressure. American Journal of Physiology-Regulatory, Integrative and Comparative Physiology. 2010;298(4):R851–R861. doi: 10.1152/ajpregu.00002.2010 20106993 PMC2853398

[55] Feraille E, Dizin E. Coordinated control of ENaC and Na+, K+-ATPase in renal collecting duct. Journal of the American Society of Nephrology. 2016;27(9):2554–2563. doi: 10.1681/ASN.2016020124 27188842 PMC5004664

[56] Laghmani K, Preisig PA, Alpern RJ. The role of endothelin in proximal tubule proton secretion and the adaptation to a chronic metabolic acidosis. Journal of nephrology. 2002;15:S75–87. 12027224

[57] Guyton A, Hall J, Coleman T, Manning R, Norman R. Hypertension: Pathophysiology, diagnosis, and management. Raven Press New York, NY, USA. 1990;.

[58] Hall J, Brands M, Shek E. Central role of the kidney and abnormal fluid volume control in hypertension. Journal of human hypertension. 1996;10(10):633–639. 9004086

[59] Hall JE. The kidney, hypertension, and obesity. Hypertension. 2003;41(3):625–633. doi: 10.1161/01.HYP.0000052314.95497.78 12623970

[60] Biemesderfer D, Reilly RF, Exner M, Igarashi P, Aronson PS. Immunocytochemical characterization of Na (+)-H+ exchanger isoform NHE-1 in rabbit kidney. American Journal of Physiology-Renal Physiology. 1992;263(5):F833–F840. doi: 10.1152/ajprenal.1992.263.5.F833 1279986

[61] Diamond JM, Bossert WH. Standing-gradient osmotic flow a mechanism for coupling of water and solute transport in epithelia. Journal of General Physiology. 1967;50(8):2061–2083. doi: 10.1085/jgp.50.8.2061 6066064 PMC2225765

[62] Guyton AC, Hall JE. Medical physiology. Gökhan N, Çavuşoğlu H (Çeviren). 2006;3.

[63] Edwards A, Layton AT. Cell Volume Regulation in the Proximal Tubule of Rat Kidney. Bull Math Biol. 2017;79(11):2512–2533. doi: 10.1007/s11538-017-0338-6 28900833 PMC5660676

[64] Goble C, Cohen-Boulakia S, Soiland-Reyes S, Garijo D, Gil Y, Crusoe MR, et al. FAIR computational workflows. Data Intelligence. 2020;2(1-2):108–121. doi: 10.1162/dint_a_00033

[65] Sarwar DM, Kalbasi R, Gennari JH, Carlson BE, Neal ML, Bono Bd, et al. Model annotation and discovery with the Physiome Model Repository. BMC Bioinformatics. 2019;20(1):457. doi: 10.1186/s12859-019-2987-y 31492098 PMC6731580

