# Supplementary material for: A modular and reusable model of epithelial transport in the proximal convoluted tubule
Source: PLoS One. 2022 Nov 10;17(11):e0275837. doi: 10.1371/journal.pone.0275837 (PMC9648790; doi:10.1371/journal.pone.0275837)
Supplement: S1 File — (ZIP) [file pone.0275837.s001.zip › Supporting Information.pdf]

Supporting information

Parameters	Membrane Properties				
	ME	MI	IE	ES	IS
	Membrane Area [cm^2/cm^2.epithelial]				
$A_{\alpha\beta}$	0.001	36.0	36.0	Eq. 61	1.0
	Water Permeability$\times RT$ [cm.s^{-1}.Osmol^{-1}]				
$L_{p\alpha\beta}$	$0.2e^{+02}$	$0.2e^{-03}$	$0.2e^{-03}$	$0.6e^{+01}$	$0.2e^{-03}$
	Permeability Coefficient, $H_{\alpha\beta}$ [cm.s^{-1}], Eq. 39				
Na^+	0.13 e^{+1}	0.0	0.39 e^{-8}	0.5 e^{-1}	1.0
K^+	0.145 e^{+1}	0.25 e^{-6}	0.2 e^{-5}	0.7 e^{-1}	0.2 e^{-5}
Cl^-	0.1 e^{+1}	0.0	0.0	0.6 e^{-1}	0.0
$HCO_3{}^-$	0.4 e^{+0}	0.1 e^{-7}	0.0	0.5 e^{-1}	0.0
$H_2CO_3{}^+$	0.4 e^{+0}	0.65 e^{-3}	0.65 e^{-3}	0.5 e^{-1}	0.65 e^{-3}
CO_2	0.4 e^{+0}	0.75 e^{-1}	0.75 e^{-1}	0.5 e^{-1}	0.75 e^{-1}
$HPO_4{}^{-2}$	0.2 e^{+0}	0.95 e^{-8}	0.225 e^{-7}	0.4 e^{-1}	0.225 e^{-7}
$H_2PO_4{}^-$	0.2 e^{+0}	0.0	0.33 e^{-6}	0.4 e^{-1}	0.33 e^{-6}
Urea	0.4 e^{+0}	0.105 e^{-5}	0.1 e^{-5}	0.8 e^{-1}	0.1 e^{-5}
NH_3	0.25 e^{+0}	0.85 e^{-3}	0.1 e^{-2}	0.2 e^{0}	0.1 e^{-2}
$NH_4{}^+$	0.25 e^{+0}	0.215 e^{-6}	0.6 e^{-6}	0.2 e^{0}	0.6 e^{-6}
H^+	0.3 e^{+2}	0.85 e^{-2}	0.85 e^{-2}	0.3 e^{+2}	0.85 e^{-2}
$HCO_2{}^-$	0.7 e^{+0}	0.0	0.19 e^{-6}	0.5 e^{-1}	0.19 e^{-6}
H_2CO_2	0.14 e^{+1}	0.5 e^{-1}	0.6 e^{-1}	0.9 e^{-1}	0.6 e^{-1}
Glucose	0.8 e^{-1}	0.0	0.75 e^{-5}	0.3 e^{-1}	0.75 e^{-5}
	Reflection Coefficient, $\sigma_{\alpha\beta}$				
Na^+	0.75	1.0	1.0	0.0	1.0
K^+	0.6	1.0	1.0	0.0	1.0
Cl^-	0.3	1.0	1.0	0.0	1.0
$HCO_3{}^-$	0.9	1.0	1.0	0.0	1.0
$H_2CO_3{}^+$	0.9	1.0	1.0	0.0	1.0
CO_2	0.9	1.0	1.0	0.0	1.0
$HPO_4{}^{-2}$	0.9	1.0	1.0	0.0	1.0
$H_2PO_4{}^-$	0.9	1.0	1.0	0.0	1.0
Urea	0.7	0.95	0.95	0.0	0.95
NH_3	0.3	0.5	0.5	0.0	0.5
$NH_4{}^+$	0.6	1.0	1.0	0.0	1.0
H	0.2	1.0	1.0	0.0	1.0
$HCO_2{}^-$	0.3	1.0	1.0	0.0	1.0
H_2CO_2	0.7	0.95	0.95	0.0	0.95
Glucose	1.0	1.0	1.0	0.0	1.0
	Coupled Transport Pathways: [mmol^2.J^{-1}.s^{-1}.cm^{-2}]				
K^+-Cl^-	-	-	0.5 e^{-8}	-	0.5 e^{-8}
Na^+-2 $HCO_3{}^-$/Cl^-	-	-	0.14 e^{-6}	-	0.14 e^{-6}
Na^+-3 $HCO_3{}^-$	-	-	0.15 e^{-7}	-	0.15 e^{-7}
Na^+/H^+	-	0.225 e^{-7}	-	-	-
Na^+/$NH_4{}^+$	-	0.15 e^{-8}	-	-	-
Na^+-glucose	-	0.75 e^{-8}	-	-	-
Na^+-$H_2PO_4{}^-$	-	0.5 e^{-8}	-	-	-
Cl^-/$HCO_2{}^-$	-	0.5 e^{-8}	-	-	-
Cl^-/$HCO_3{}^-$	-	0.2 e^{-8}	-	-	-
	Active Transport: [mmol^2.J^{-1}.s^{-1}.cm^{-2}]				
H^+	-	Eq. 53	-	-	-
$Na^+K/^+$-ATPS	-	-	Eq. 58	-	Eq. 58

Continued on next page

Table 1 – continued from previous page

Parameters	Membrane Properties				
	ME	MI	IE	ES	IS
	Compartment Properties				
	M	E	I	S	
	Concentration, $C_\alpha(i)$[mol/l] & Valence Number, Z_i				
Na^+	0.14	0.14	0.02	0.14	1
K^+	0.49 e^{-2}	0.46 e^{-2}	0.13	0.49 e^{-2}	1
Cl^-	0.113	0.11	0.16 e^{-1}	0.113	-1
$HCO_3{}^-$	0.024	0.25	0.25 e^{-1}	0.024	-1
H_2CO_3	0.44 e^{-5}	0.4 e^{-5}	0.43 e^{-5}	0.44 e^{-5}	0
CO_2	0.15 e^{-2}	0.14 e^{-2}	0.14 e^{-2}	0.15 e^{-2}	0
$HPO_4{}^{-2}$	0.2 e^{-2}	0.86 e^{-3}	0.94 e^{-2}	0.2 e^{-2}	-2
$H_2PO_4{}^-$	0.927 e^{-3}	0.28 e^{-2}	0.27 e^{-2}	0.927 e^{-3}	-1
CH_4N_2O	0.5 e^{-2}	0.4 e^{-2}	0.4 e^{-2}	0.5 e^{-2}	0
NH_3	0.282 e^{-5}	0.26 e^{-5}	0.35 e^{-5}	0.282 e^{-5}	0
$NH_4{}^+$	0.197 e^{-3}	0.17 e^{-3}	0.23 e^{-3}	0.197 e^{-3}	1
$HCO_2{}^-$	0.1 e^{-2}	0.7 e^{-3}	0.5 e^{-3}	0.1 e^{-2}	-1
H_2CO_2	0.285 e^{-6}	0.2 e^{-6}	0.9 e^{-7}	0.285 e^{-6}	0
$C_6H_{12}O_6$	0.5 e^{-2}	0.7 e^{-2}	0.1 e^{-1}	0.5 e^{-2}	0
Impermeant	0.0	0.0	Variable, Eq. 60	0.002	-1
Osmolality	298.8	304	301	298.8	
	Electrical Potential [mV]				
ψ_α	Variable, Eq. 39	Variable	Variable	0.0	
	Hydrostatic Pressure [mmHg]				
P_α	15.0	Variable, Eq. 37	15	0.0	
	Volume [cm^3/cm^2. epithelial]				
V_α	Variable Eq. 62	Variable Eq.60	-	-	

The table can be categorised to two different parts: first, membrane properties (ME, MI, IE, ES,IS); then, compartment properties (M, E, I, S).

Table 2: W-PCT-E Model's Constant Parameters.

Constant Parameters in PCT-Epithelial model			
Constant	Definition	Unit	Value
R	Gas Const	J/mol.K	8.314
T	Temp	K	273.15
F	Faraday	C/mol	96.5 e^3
K_h	Hydration Const CO_2	1/s	1.45 e^3
K_d	Dehydration Const CO_2	1/s	4.96 e^5
η	Fluid Viscosity	mmHg/s	6.4 e^{-6}
$P(HCO_3{}^-)$	pK of $HCO_3{}^-$	–	3.57
$P(HCO_2{}^-)$	pK of $HCO_2{}^-$	–	3.76
$P(NH_3)$	pK of NH_3	–	9.15
$P(HPO_4{}^-)$	pK of $HPO_4{}^{-2}$	–	6.8
$P(Buf^-)$	pK of Cell Buffers	–	7.5
V_{I0}	Reference Cell Volume	cm^3/cm^2.epithelium	0.1 e^{-2}
C_{IMP0}	Reference Cell Impermeant	mol/l	0.6 e^{-1}
C_{TBUF}	Total Cell Buffer Conc	mmol/ml	0.6 e^{-1}
$Q_{NH_4^+}$	Ammonia generation	mmol/s.cm^2	$0.7e-7$

Here, $pK = \log(\frac{1}{K_\alpha})$ and K_α represents the equilibrium constant for different buffer pairs. In some cases, we use $RT = 1.93\ e^4$ mmHg.ml/mmol and in some other cases $RT = 2.57$ J/mmol just to simplify the calculations.

We need to highlight that in here $C_{TBUF} = C_{BUF} + C_{HBUF}$, C_{BUF} and C_{HBUF} are variables and they indicate the cell buffer concentration and protonated cell buffer concentration, respectively.

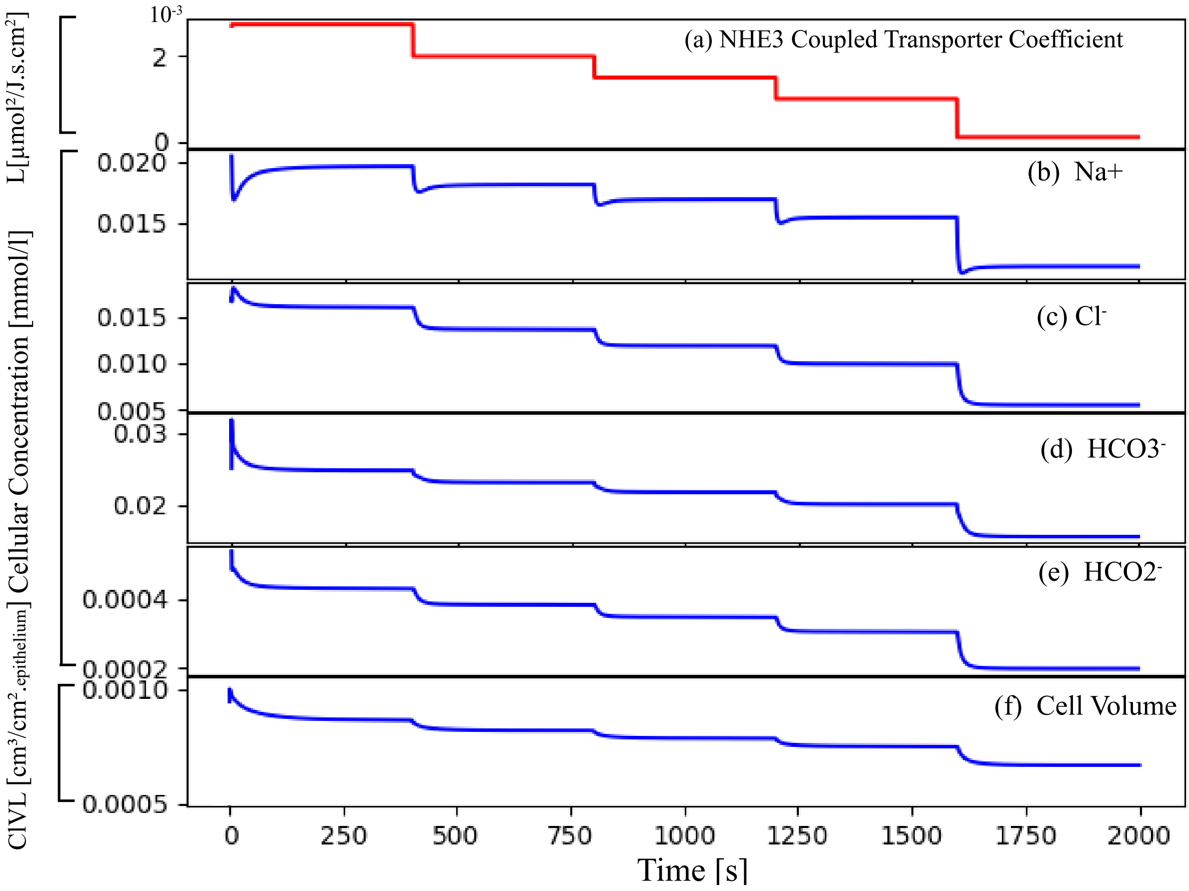

Figure 1: Effect of reduction in NHE3 coupled transporter coefficient on the cellular concentration and cell volumes. Panel (a): NHE3 coupled transporter coefficient decreased in a step-wise manner, at each step there is an decrease of 20% in regard to the original value. Panels (b)-(e): represent changes in some selected cellular solutes concentrations due to the changes in NHE3 coupled transporter coefficient. Panel (f): changes on cell volume.

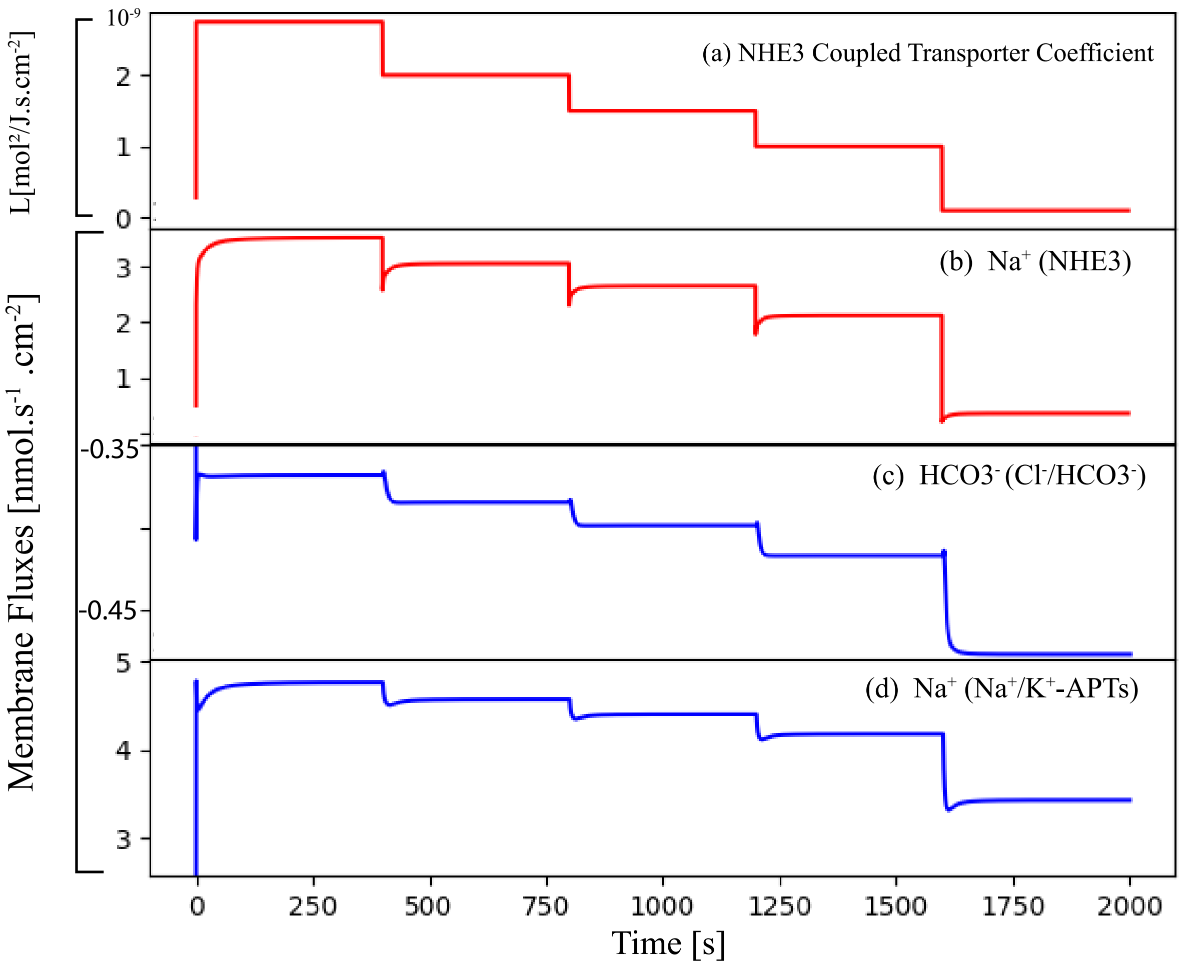

Figure 2: Effect of reduction in NHE3 coupled transporter coefficient on the membrane fluxes. Panel (a): NHE3 coupled transporter coefficient decreased in a step-wise manner, at each step there is an decrease of 20% in regard to the original value. Panels (b)-(d): represent changes in some selected membrane fluxes for selected transporters due to the changes in NHE3 coupled transporter coefficient.

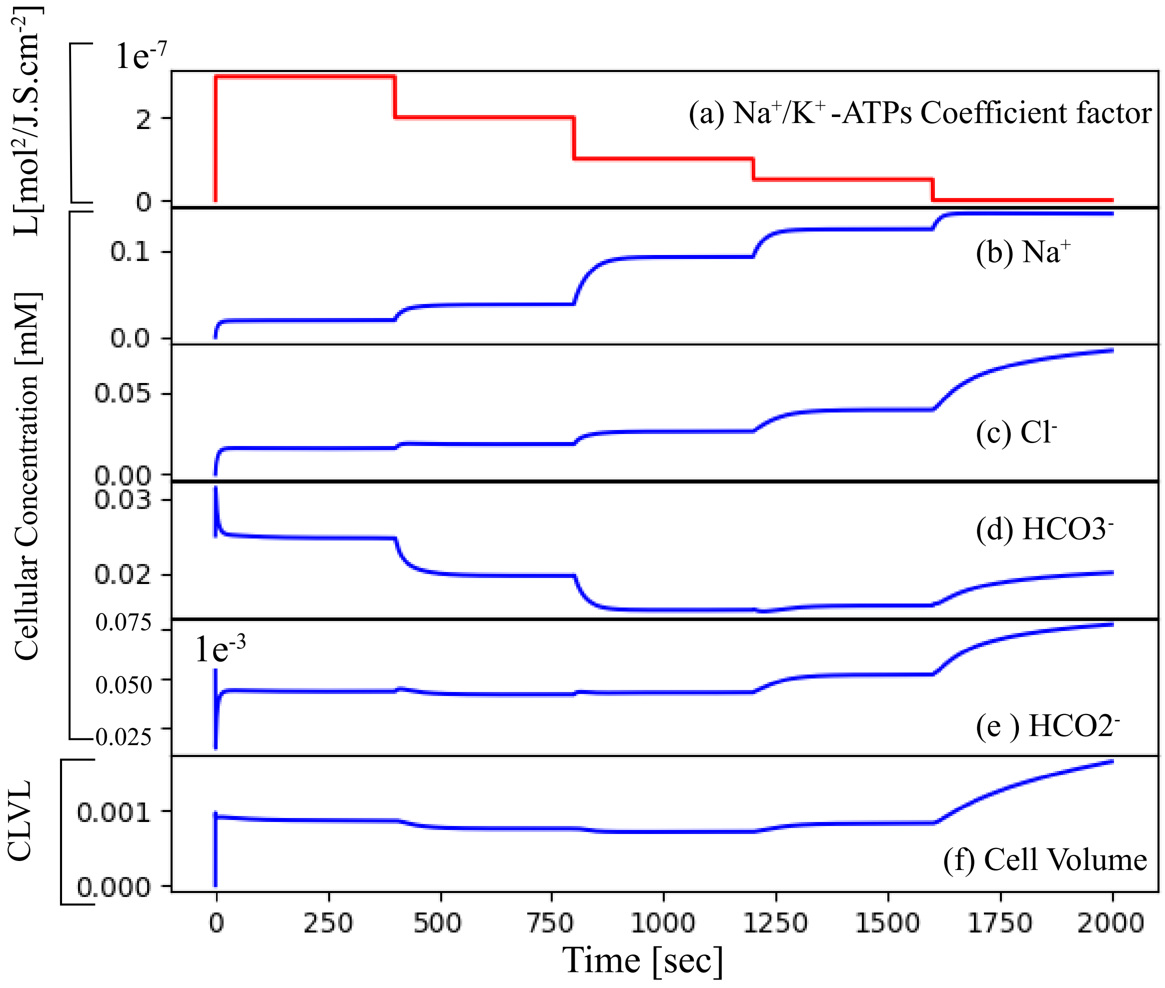

Figure 3: Effect of reduction in Na^+/K^+-ATPS coefficient on the cellular concentration and cell volumes. Panel (a): Na^+/K^+-ATPS coefficient factor decreased in a step-wise manner, from the maxiumum value of $3.0e^{-7}$ to zero. Panels (b)-(e): represent changes in some selected cellular solutes concentrations due to the changes in Na^+/K^+-ATPS coefficient factor. Panel (f): changes on cell volume.

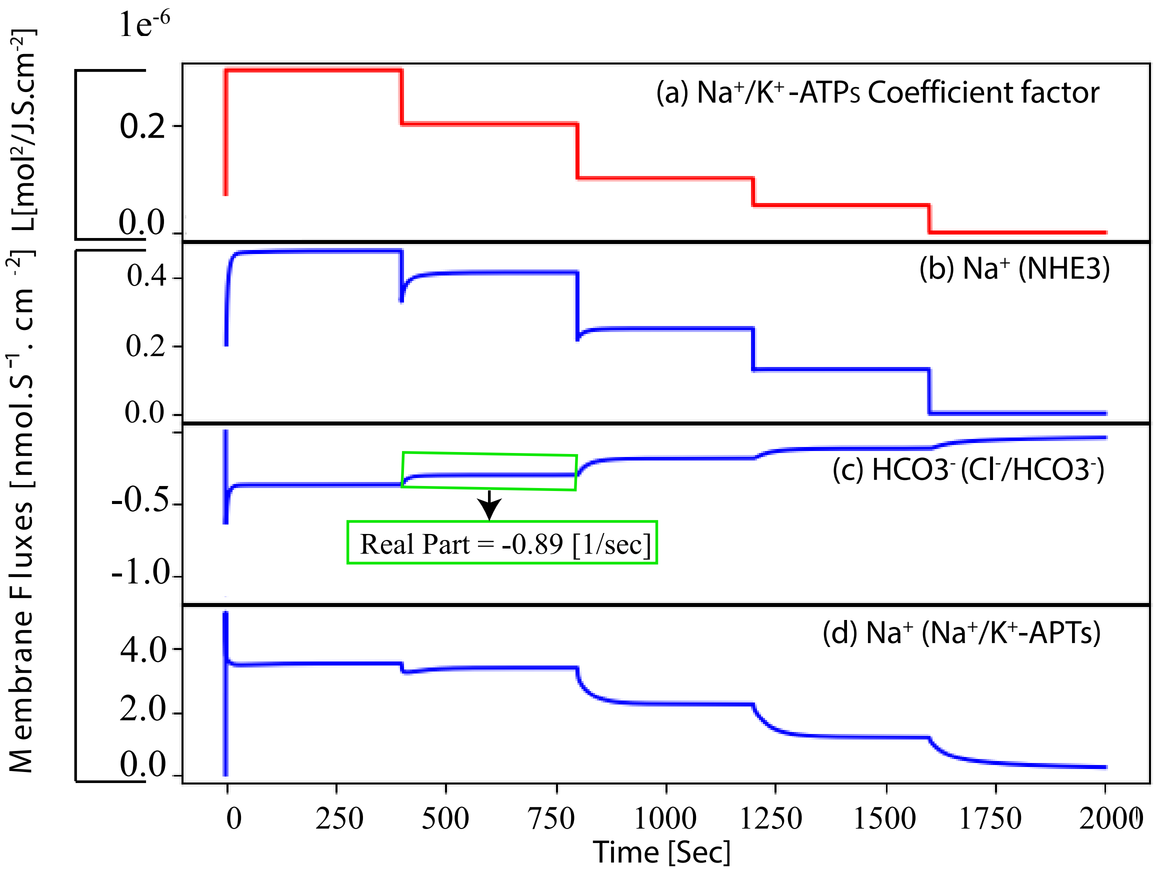

Figure 4: Effect of reduction in NHE3 coupled transporter coefficient on the membrane fluxes. Panel (a): Na^+/K^+-ATPS coefficient parameter decreased in a step-wise manner, from the maxiumum value of $3.0e^{-7}$ to zero. Panels (b)-(d): represent changes in some selected membrane fluxes for selected transporters due to the changes in Na^+/K^+-ATPS coefficient factor. To aid clarity, the green rectangle in subplot (c) indicates the simulations' time frame that we choose to investigate the system stability. We calculate the eigenvalues and then extract the eigenvalue with the maximum real part; the negative values indicate a stable system.
